# Myosin V: Chemomechanical-coupling ratchet with load-induced mechanical slip

**DOI:** 10.1038/s41598-017-13661-0

**Published:** 2017-10-18

**Authors:** Tomonari Sumi

**Affiliations:** 1 Research Institute for Interdisciplinary Science, Okayama University, 3-1-1 Tsushima-Naka, Kita-ku, Okayama, 700-8530 Japan; 2 Department of Chemistry, Faculty of Science, Okayama University, 3-1-1 Tsushima-Naka, Kita-ku, Okayama, 700-8530 Japan

## Abstract

A chemomechanical-network model for myosin V is presented on the basis of both the nucleotide-dependent binding affinity of the head to an actin filament (AF) and asymmetries and similarity relations among the chemical transitions due to an intramolecular strain of the leading and trailing heads. The model allows for branched chemomechanical cycles and takes into account not only two different force-generating mechanical transitions between states wherein the leading head is strongly bound and the trailing head is weakly bound to the AF but also load-induced mechanical-slip transitions between states in which both heads are strongly bound. The latter is supported by the fact that ATP-independent high-speed backward stepping has been observed for myosin V, although such motility has never been for kinesin. The network model appears as follows: (1) the high chemomechanical-coupling ratio between forward step and ATP hydrolysis is achieved even at low ATP concentrations by the dual mechanical transitions; (2) the forward stepping at high ATP concentrations is explained by the front head-gating mechanism wherein the power stroke is triggered by the inorganic-phosphate (Pi) release from the leading head; (3) the ATP-binding or hydrolyzed ADP.Pi-binding leading head produces a stable binding to the AF, especially against backward loading.

## Introduction

Myosin V, a member of the myosin superfamily, is a two-headed molecular motor that transports intracellular cargos along a network of actin filaments using the free energy caused by ATP hydrolysis^1–5^. It moves processively and directionally towards the plus end of an actin filament, taking 36-nm steps by alternately swinging forward two lever arms, namely, in a walking-like or hand-over-hand manner^6–10^. The myosin-V movement can be interpreted on the basis of (a) the binding affinity of the head to the actin filament (AF) that depends upon nucleotide binding to the head and (b) the difference between the chemical transition rates in the leading and trailing heads that is regulated by an intramolecular strain of each head^11–15^. Based on these fundamental observations, several models^6,12,16–23^ have been proposed to characterize the motor dynamics. The standard model of myosin V with only the single kinetic cycle is as follows: it is strongly bound to the AF using both heads with ADP binding; after the release of ADP from the trailing head, the ATP binding to the head induces the detachment of the trailing head from the AF, resulting in the forward step. It is believed that the ADP release from the leading head in the two-head-bound state is basically prevented by the intramolecular strain of the leading and the ADP-binding leading head is necessary for causing the power stroke of the leading head^12–14^. However, we believe that a rate-limiting transition in the kinetic cycle depends upon both ATP concentration and external load and also that molecular motors might use various chemical-transition pathways to achieve robust motor properties against changes in the external environment. Thus, an excessive reduction of the chemical- and mechanical-transition pathways from the network would result in an artificial description on the chemomechanical coupling of the molecular motors, even if the simple model with such an extensive reduction quantitatively describes part of the experimental data.

Stochastic modelling with either a single working cycle^6,12,16^ or branched kinetic cycles^18–23^ has been applied to the investigation of chemical and mechanical properties of myosin V. These models basically impose restrictions on the chemomechanical cycles so that only pre-selected chemical and mechanical transitions are taken into account, in order to reduce unknown parameters for the transition rates. In the present study, introducing only as few restrictions on the working cycles as possible, we apply a network representation of kinetic pathways based on a chemomechanical network theory proposed by Liepelt and Lipowsky^22,24–26^ and characterize nucleotide-dependent and load-dependent main working cycles of forward and backward stepping. In our modelling, we take into account all possible mechanical transitions between states not only in which one head is strongly bound and the other head is weakly bound to the AF, but also in which both heads are strongly bound to the AF. The former is a necessary condition for producing a force-generating mechanical step, whereas the latter is also required in order to describe high-speed processive backward stepping induced by superstall loading which has been experimentally observed as a remarkable motor property of myosin V^27^, whereas the high-speed backward stepping has never been observed in the case of kinesin. The unified description for myosin V’s motility upon both the force-generating mechanical step and high-speed backward step remains as an open question. The velocity of backward movement under superstall loading does not greatly depend upon ATP concentration so as myosin V seems to act as a load-induced slipping ratchet as well, whereas that of the load-assisted forward movement obviously increases along with this concentration^27^. These experimental observations suggest the importance of the mechanical transitions that do not directly couple with the chemical transitions, in addition to the force-generating mechanical transitions, in the case of myosin V. Furthermore, with consideration of the intramolecular strain of the leading and trailing heads, which would be interpreted as the difference in the catalytic-domain conformations between of the leading and trailing heads with pre-recovery-stroke and post-recovery-stroke conformations, respectively we also introduce the asymmetries and similarity relations between the chemical transitions as we did for kinesin^28^, in order to systematically reduce the number of unknown chemical-transition rates. Our network model for myosin V provides quantitative description on the single-molecule experimental results at various ATP and ADP concentrations concerning external-load dependence of the motor velocity, the ratio of forward steps to backward steps as a function of the external load, the mean run length, and the detachment rate from the AF, using only one set of the chemical- and mechanical-transition rates. Finally, we conclude the common and different characteristics of the motor properties of myosin V and kinesin from the chemomechanical network point of view.

## Chemomechanical network modelling of molecular motor myosin V

### Myosin V’s state space

**Figure 1:**
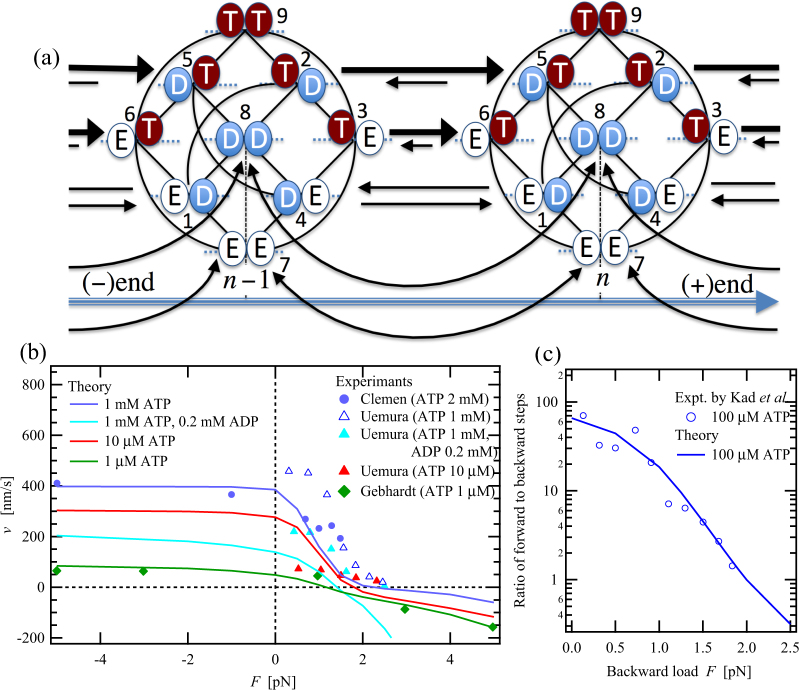
(**a**) A nine-state chemomechanical network model of myosin V based on a network representation consisting of 3^2^ = 9 full chemical states connected by eighteen chemical and five mechanical transitions. The figure shows that the nine chemical states are periodically repeated along an actin filament (AF) with a spacing of *l* = 36 nm. The myosin-V’s heads with bound ATP or ADP are denoted by T and D, respectively, while an empty head is denoted by E. Both the D and E states are strongly bound to the AF, while the T state is basically weakly bound^11^. The vertical displacement of the heads with the T state from the base line indicates the weak binding of the T heads. The solid lines indicate the chemical transitions and those with arrows indicate the mechanical transitions. To calculate the mean run length [Fig. 3b,e] and the detachment rate of myosin V from the AF [Fig. 3c,f], we extend the nine-state model while taking account of the detachment transitions of myosin V from states 9 (TT), 5 (DT), 2 (TD), 6 (ET), 8 (DD), 1 (ED) and 7 (EE) [see Calculation details and the Supplementary Information]. (**b**) External-load dependences of the motor velocity at several ATP and ADP concentrations. (**c**) External-load dependence of the ratio of the numbers between the forward and backward steps. The experimental data shown by symbols is obtained from the literature^19,21,27,45^.

To describe the dynamics of molecular-motor myosin V, we apply the chemomechanical network theory presented by Liepelt and Lipowsky^22,24–26^, according to which the catalytic cycle of ATPase with a single catalytic domain for each head is modelled using three chemical states in which the catalytic domain is empty (E), occupied by ATP (T), or occupied by ADP (D). In several kinetic models for myosin V, a chemical state in which the catalytic domain is occupied by the combination of ADP and inorganic phosphate (Pi) is also taken into account and thus the catalytic cycle of the ATPase consists of the four chemical states, ATP, ADP.Pi, ADP and empty^18,20,21,23,29^. On the other hand, in our modelling, it is supposed that the ADP.Pi combination state is included in the ATP state. Thus, the catalytic cycle of myosin V, a two-headed molecular motor with one catalytic domain per head, can be expressed using a network representation with 3^2^ = 9 different chemical states, as shown in Fig. 1a. The network representation based on the nine-state space provides a good starting point for our systematic modelling on a chemomechanical coupling in the two-headed motors. It has been experimentally demonstrated that the binding affinity of myosin V’s head to an AF depends upon the chemical state of the head: the head in the states D and E is strongly bound to the AF whereas that in the state T is weakly bound to it^11^. In Fig. 1a, the nucleotide-state-dependent binding affinity of each head is schematically indicated as its vertical displacement from the dotted base line.

### Mechanical-step transitions

If myosin V walks in the hand-over-hand manner^6–10^ by converting ATP-hydrolysis energy into a mechanical work, the leading head and the trailing head exchange their position on an AF during mechanical-step transitions, implying that the leading head should be strongly bound to the AF, while the trailing head should be easily unbound from the AF. In the full nine-state-network representation, this requirement for a force-generating mechanical step leads to two mechanical transitions from state 2 (TD) to state 5 (DT) and from state 3 (TE) to state 6 (ET) [see Fig. 1a]. Recently, Kodera *et al*. applied a new atomic-force microscopy (AFM) technique to cause detachment from an AF of myosin V’s trailing head in the two-head-bound state in the absence of nucleotides as well as in the presence of ADP. They observed that myosin V almost always caused forward steps with a step size of 36 nm, whereas it moved neither backwards nor forwards when the leading head was detached^30^. This remarkable observation indicates that neither ADP binding to the leading head nor ATP binding to the trailing head is always necessary to produce a forward step. We, therefore, assume in this study that not only the transition from states 2 (TD) to 5 (DT) but also that from states 3 (TE) to 6 (ET) can produce a force-generating forward step. How transitions between states 3 (TE) and 6 (ET) affect the chemomechanical coupling and motor dynamics of myosin V has hardly been investigated yet.

As one of the extraordinary motor properties of myosin V, high-speed processive backward stepping induced by superstall loading has been observed. Although the velocity of the load-assisting forward stepping obviously increases along with ATP concentration, that of the load-induced backward stepping does not greatly depend upon this concentration^27^. Thus, we equally take into account all possible mechanical transitions between states in which both heads are strongly bound to the AF, i.e. the transitions between states 8 (DD) and 8 (DD), between states 1 (ED) and 4 (DE), and between states 7 (EE) and 7 (EE), in addition to the force-generating mechanical transitions. The 8-to-8 and 7-to-7 transitions do not directly couple with the chemical states and thus would play an important role in the ATP-independent high-speed backward motility under superstall loading.

### A nine-state model

Bierbaum and Lipowsky^22^ proposed a six-state chemomechanical network model for myosin V, wherein states 3 (TE), 4 (DE) and 9 (TT) were omitted from the full nine-state representation and only the mechanical transitions between states 2 (TD) and 5 (DT) and between states 7 (EE) and 7 (EE) were taken into account. They demonstrated that it could describe the experimental data for external-load dependences of motor velocity and ratio of forward steps to backward steps, and also for nucleotide-concentration dependences of motor velocity and mean run length^22^. They also showed that the six-state model could describe the high-speed backward motility induced by superstall loading. However, according to the similarity relations among the chemical transitions based on the effects of the intramolecular strain of the leading and trailing heads, such as the difference in the catalytic-domain conformations between of the leading and trailing heads with the post-recovery-stroke and pre-recovery stroke conformations^31^, respectively, we find internal inconsistencies among the transition rates have been introduced tacitly in this model [see Fig. S1 in the Supplementary Information]. Zhang *et al*.^23^ also proposed a six-state chemomechanical network model for myosin V, wherein states 3 (TE), 4 (DE) and 7 (EE) were omitted from the full nine-state representation and only the mechanical transitions between states 2 (TD) and 5 (DT) were taken into account. Although no obvious internal inconsistencies (as seen in the six-state model by Bierbaum and Lipowsky^22^) are found in this network structure, we do notice an inconsistency between the transition rates where they do not satisfy the similarity relations between the chemical transitions on the basis of the intramolecular strain of the leading and trailing heads [see Fig. S2 in the Supplementary Information]. In addition, the load dependences of the motor properties have not been examined in this model.

Our motivation in the present study is to clarify the main working cycles that drive the forward and backward-stepping motilities of myosin V and also the dependences of the main working cycles on both ATP concentration and external load on the basis of chemomechanical network modelling without intuitive pre-selection of the state space and transition pathways. For this purpose, we finally attained to a nine-state chemomechanical network model shown in Fig. 1a. In this model, all of the chemical states, possible chemical and mechanical transitions other than the 9-to-9 transition are taken into account. The necessity of this nine-state model can be verified by the asymmetries and similarity relations between the chemical transitions, which have been introduced in our previous study on kinesin^28^. The asymmetries between the chemical transitions taking place in the leading and trailing heads are caused by the difference in the catalytic-domain conformations between of the leading and trailing heads with the post-recovery-stroke and pre-recovery-stroke conformations, respectively, namely, the intramolecular strain of the leading and trailing heads. In the same way, the similarity relations between the chemical transitions in the leading and trailing heads are also obtained based on this intramolecular strain. For instance, the ATP hydrolysis or Pi release in the leading head, i.e. the 5-to-8 transition, should be similar to the 6-to-1 transition. The ATP binding to the trailing head, the 1-to-2 transition, should be similar to the 7-to-3 and the 6-to-9 transitions as well. Although the leading head is slightly weakly bound to the AF during the 6-to-9 transition compared with the 1-to-2 and 7-to-3 transitions, the 6-to-9 transition should be more similar to the 1-to-2 and 7-to-3 transitions than the 3-to-9 transition. It is because, the trailing head to which ATP is going to bind during the 6-to-9 transition has the pre-recovery-stroke conformation and the trailing head during both the 1-to-2 and 7-to-3 transitions has, in the same way, the pre-recovery-stroke one, whereas the trailing head to which ATP is going to bind during the 3-to-9 transition has the post-recovery-stroke one. Adapting these similarity relations among the chemical transitions, we can reduce the number of unknown chemical-transition rates in the nine-state network. All of the similarity relations between the chemical transitions introduced in the present study are summarized in the Supplementary Information.

## Results

### Load dependence of the motor properties

**Figure 2:**
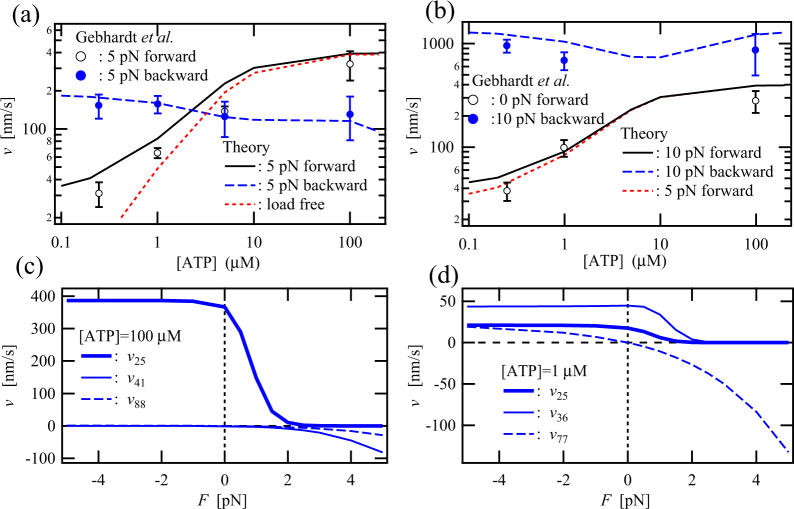
ATP-concentration dependences of the motor velocities, *v*, for forward and backward movements at (**a**) 5-pN and (**b**) 10-pN forward and backward loads. For comparison, the motor velocities at 0-pN and 5-pN forward loads are also shown in (**a**) and (**b**), respectively. The experimental data indicated by symbols are obtained from the literature^27^. (**c**) and (**d**) show the main contributions to the total velocity caused by each mechanical transition as a function of external load at ATP concentrations of 100 µM and 1 µM, respectively.

Figure 1b shows external-load dependences of the motor velocity *v* at several ATP concentrations. As the backward load increases, the motor velocity *v* decreases and myosin V is stalled by a backward load called a stall load. The stall load for myosin V depends upon ATP concentration, while that for kinesin does not^32^. Under a superstall load, i.e. a backward load larger than the stall load, myosin V can processively move at high speed and the backward velocity monotonically increases as the external load increases^27^. The high-speed processive backward motility and its large load dependence are never observed in the case of kinesin^32^. In contrast to the backward velocity under superstall loading, the forward velocity does not depend on the assisting load, especially at high ATP concentrations. The almost-constant velocities at varying assisting loads have been observed in kinesin as well^32,33^. In addition, the forward velocities at constant assisting loads depend more strongly on ATP concentration than do the backward velocities at constant superstall loads, except in the case where ADP is added. The similar strong ATP-concentration dependence of the load-assisted forward velocity has been observed in the case of kinesin as well^32,34^. These observations for myosin V imply that the forward-stepping cycle at the high ATP concentration strongly couples with ATP hydrolysis and that the hydrolysis cycle of ATP is a rate-limiting process, even though the 8-to-8 and 7-to-7 transitions, which do not directly couple with the nucleotide state of the heads, are also introduced in the nine-state model. On the other hand, the remarkable external-load dependence of the backward velocity under superstall loading could be interpreted by a large contribution to the total velocity from the 8-to-8 and/or 7-to-7 transitions. The backward and forward motilities forced by high external loading will be discussed in detail in Fig. 2.

Figure 1c shows the ratio between the numbers of forward and backward steps as a function of external load at the ATP concentration of 100 µM. The nine-state model can quantitatively describe the external-load dependence of the ratio of forward steps to backward steps. To achieve the high ratio around 60 under the load-free condition, the main forward-stepping cycle at 100-µM ATP would have to avoid states 8 (DD) and 7 (EE). It is because, if states 8 (DD) and 7 (EE) frequently appeared in the main forward cycle, the 8-to-8 and 7-to-7 transitions that cause symmetric random walk in the absence of external load would significantly lower the ratio of forward steps to backward steps. The main forward-stepping cycle will be discussed in detail later on.

### ATP-concentration dependence of the forward and backward velocities under loading

Figure 2a,b show ATP-concentration dependences of the motor velocity *v* for forward and backward stepping forced by forward and backward loads of (a) 5 pN and (b) 10 pN. We find that the forward velocity at high ATP concentrations is not affected by the strength of the forward loading in comparison between the black solid and red dotted lines shown in Fig. 2a,b; however, it does strongly depend upon ATP concentration, as shown by the black solid lines in these figures. Concerning the ATP-dependent velocity under the assisting loading, the main transition pathway causing forward motility depends upon ATP concentration, as shown in Fig. 2c,d: the main mechanical transition working at 100-µM ATP is the 2-to-5 transition [Fig. 2c], while the largest and second-largest contributions to the total velocity at 1-µM ATP are the 3-to-6 and 2-to-5 transitions, respectively [Fig. 2d]. These mechanical transitions directly couple with the nucleotide state of the heads, because these nucleotide states are exchanged by the mechanical step [see Fig. 1a]. Thus, the rate-limiting process of the load-assisting forward cycle is basically attributable to ATP-hydrolysis processes. This is why the forward velocity strongly depends upon the ATP concentration but is hardly affected by the assisting load.

We can also see that the backward velocity is significantly increased by the increase in backward load from 5 to 10 pN in comparison between the blue broken lines shown in Fig. 2a,b, whereas the backward velocity under backward loading is less affected by ATP concentration than the forward velocity under assisting loading. However, the contributions to the backward velocity generated by each mechanical transition are completely different at the high- and low-ATP concentrations, as shown in Fig. 2c,d: under backward loading by 5 pN, the 1-to-4 and 8-to-8 transitions mainly contribute to the backward velocity at 100-µM ATP [Fig. 2c], while the 7-to-7 transition predominantly contributes to the backward velocity at 1-µM ATP [Fig. 2d]. Neither the 8-to-8 nor 7-to-7 transitions directly couples with ATP hydrolysis processes, but are basically caused by external loading. Therefore, the high-speed backward motility is less sensitive to ATP concentration than the forward motility but strongly depends on the strength of backward load. These results indicate that myosin V acts as the load-induced-slipping ratchet as well as the chemomechanical-coupling ratchet.

### Nucleotide-concentration-dependent motor properties without external loading

**Figure 3:**
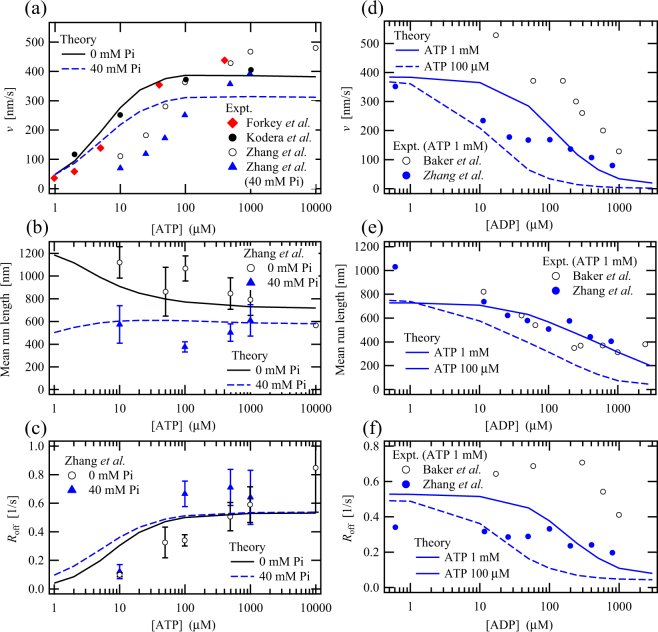
ATP-concentration dependences of (**a**) the motor velocity *v*, (**b**) the mean run length until myosin V detaches from an actin filament and (**c**) the detachment rate of myosin V, at Pi concentrations of 0 and 40 mM; ADP-concentration dependences of (**d**) the motor velocity *v*, (**e**) the mean run length and (**f**) the detachment rate, at ATP concentrations of 100 µM and 1 mM. The experimental data shown by symbols are obtained from the literatures^10,18,23,35^.

Figure 3a shows ATP-concentration dependences of the motor velocity *v* at Pi concentrations of 0 and 40 mM. As ATP concentration increase, the motor velocity *v* rapidly increases until becoming saturated at ATP concentrations above about 100 µM^10,35^. In both the experiment by Zhang *et al*.^23^ and the theoretical results, the motor velocity *v* is reduced by the addition of 40-mM Pi. Figure 3b shows the ATP-concentration dependences of the mean run length until myosin V detaches from an AF. This mean run length is almost constant at the Pi concentration of 40 µM, but decreases under the Pi-free condition with increasing ATP concentration^23^, even although the motor velocity *v* monotonically increases as ATP concentration increases [Fig. 3a]. This is not the case for kinesin^36^; its mean run length increases according to an increase in the motor velocity with increasing ATP concentration. These results indicate that the detachment rate of myosin V would be increased by the increase in ATP concentration, as shown in Fig. 3c. We examined an eight-state model formed by omitting state 9 (TT) from the nine-state model [see Fig. 1a] to describe the experimental data shown in Figs 1, 2 and 3. The results were almost comparable to those of the nine-state model in that the experimental data except for several crucial motor properties could be described by the eight-state model, whereas it was found that the decrease in the mean run length as shown in Fig. 3b and the increase in the detachment rate shown in Fig. 3c could not be described by the eight-state model. Here, it is expected that states 9 (TT), 2 (TD) and 5 (DT) would frequently appear at high ATP concentrations and that myosin V staying in these states would likely detach from the AF; it is because, it would have at least one ATP-binding head that is weakly bound to the AF. These observations imply that, as the probabilities of states 9 (TT), 5 (DT) and 2 (TD) increase along with ATP concentration, so too does the frequency of detachment of myosin V via those states.

In Fig. 3d, we find a decrease in the motor velocity *v* as ADP concentration increases. Figure 3f shows that the theoretical result for the detachment rate decreases with increasing ADP concentration and that the experimental results either slightly decrease or stay almost constant. In contrast to the ATP-concentration dependence of the mean run length, the decrease in the mean run length with the increase in ADP concentration seen in Fig. 3e is attributable to the decrease in the motor velocity *v* shown in Fig. 3d.

### Efficiency of the transduction of ATP-hydrolysis free energy into mechanical work

**Figure 4:**
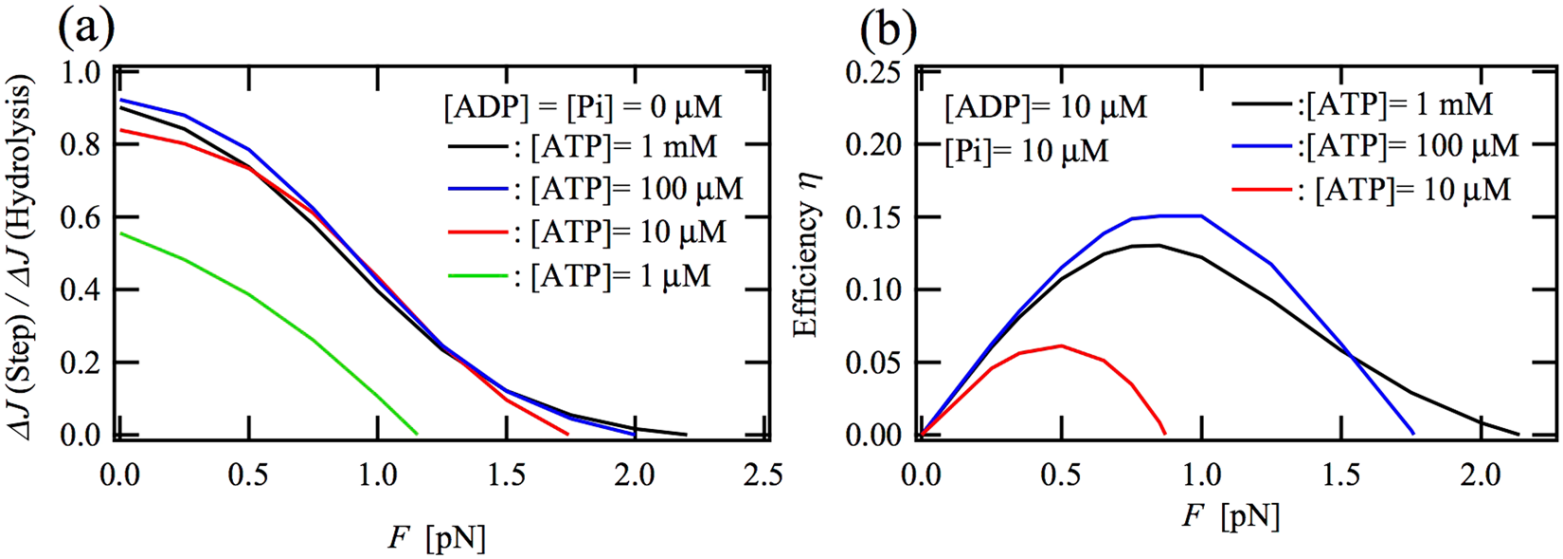
Load dependences of (**a**) the ratio of the number of forward steps to the number of ATP molecules undergoing hydrolysis, $\Delta J({\rm{Step}})/\Delta J({\rm{hydrolysis}})$, i.e. chemomechanical-coupling efficiency and (**b**) chemomechanical-transduction efficiency $\eta =Fv/[\Delta \mu \Delta J({\rm{hydrolysis}})]$ at several ATP concentrations, where *Δμ* is the difference between the chemical potentials for one molecule of ATP and for one molecule of ADP plus one molecule of Pi, i.e. $\Delta \mu ={k}_{{\rm{B}}}T\,\mathrm{ln}\{{K}_{eq}[{\rm{ATP}}]/[{\rm{ADP}}][{\rm{Pi}}]\}$ ^37^, with the equilibrium constant *K*_*eq*_ = 4.9 × 10^11^ µM^38^; $\Delta J({\rm{hydrolysis}})$ is the total excess flux arising from all the ATP hydrolysis, and $\Delta J({\rm{Step}})$ is the total excess flux provided by all of the mechanical transitions.

Figure 4a shows external-load dependences of the ratio of the number of forward steps to the amount of ATP hydrolysis, *ΔJ*(Step)/*ΔJ*(hydrolysis), i.e. chemomechanical-coupling efficiency, at several ATP concentrations in the absence of ADP and Pi, where *ΔJ*(Step) and *ΔJ*(hydrolysis) are defined as the sums of *ΔJ*_*ij*_ [shown by Eq. (2)] that are related to the mechanical-step transitions and ATP-hydrolysis transitions, respectively. Except for the lowest ATP concentration of 1 µM, the ratios under zero loading are larger than 0.8, indicating that a high ratio of transduction from the hydrolysis of one ATP molecule to one forward step is achieved, even at the low ATP concentration of 10 µM. In addition, these load-dependent curves at ATP concentrations between 10 µM and 1 mM almost overlap with each other. These results show that myosin V has robustness against changes in ATP concentration. As later be discussed based on the main working cycles, the high chemomechanical-coupling efficiency under the load-free condition would be attributable to the dual force-generating mechanical transitions, i.e. the 2-to-5 and 3-to-6 transitions.

Figure 4b shows load dependences of chemomechanical-transduction efficiencies, *η*, at ATP concentrations of 1 mM, 10 µM and 1 µM under the constant ADP and Pi concentration of 10 µM. The chemomechanical-transduction efficiency *η* is defined by $\eta =Fv/[\Delta \mu \Delta J({\rm{hydrolysis}})]$, where *Δμ* is the difference between the chemical potentials for one ATP and for one ADP plus one Pi, i.e. $\Delta \mu ={k}_{{\rm{B}}}T\,\mathrm{ln}\{{K}_{eq}[{\rm{ATP}}]/[{\rm{ADP}}][{\rm{Pi}}]\}$ ^37^, with the equilibrium constant *K*_*eq*_ = 4.9 × 10^11^ µM^38^. Under the case that the ADP and Pi concentrations are 10 µM, the maximum efficiency of myosin V at 1-mM ATP is obtained at a backward load around 0.8 pN, which is lower than the value for kinesin about 3.5 pN^28^, while the maximum *η* value for myosin V, about 0.13, is almost identical to that for kinesin. The loss in the transduction of the hydrolysis free-energy *Δμ* into the mechanical work should be attributable to both heat that irreversibly dissipates through the viscous friction of the probe^39^ and that irreversibly dissipates from the molecular motor due to chemical and mechanical slip cycles. A larger maximum value of *η* at 100-µM ATP than that at 1-mM ATP is due to the smaller value of *Δμ* at the ATP concentration of 100 µM than that at 1 mM, because *Δμ*, which appears in the denominator of $\eta =Fv/[\Delta \mu \Delta J({\rm{hydrolysis}})]$, is decreased by the decrease in ATP concentration. In the same manner, the maximum value of *η* can be slightly increased by appropriately increasing ADP and/or Pi concentrations, because *Δμ* is decreased by an increase in the concentrations of ADP and/or Pi if the motor velocity *v* is not mostly decreased by the increase in the ADP and/or Pi concentrations.

### Chemical-transition pathways at different ATP concentrations and external loads

**Figure 5:**
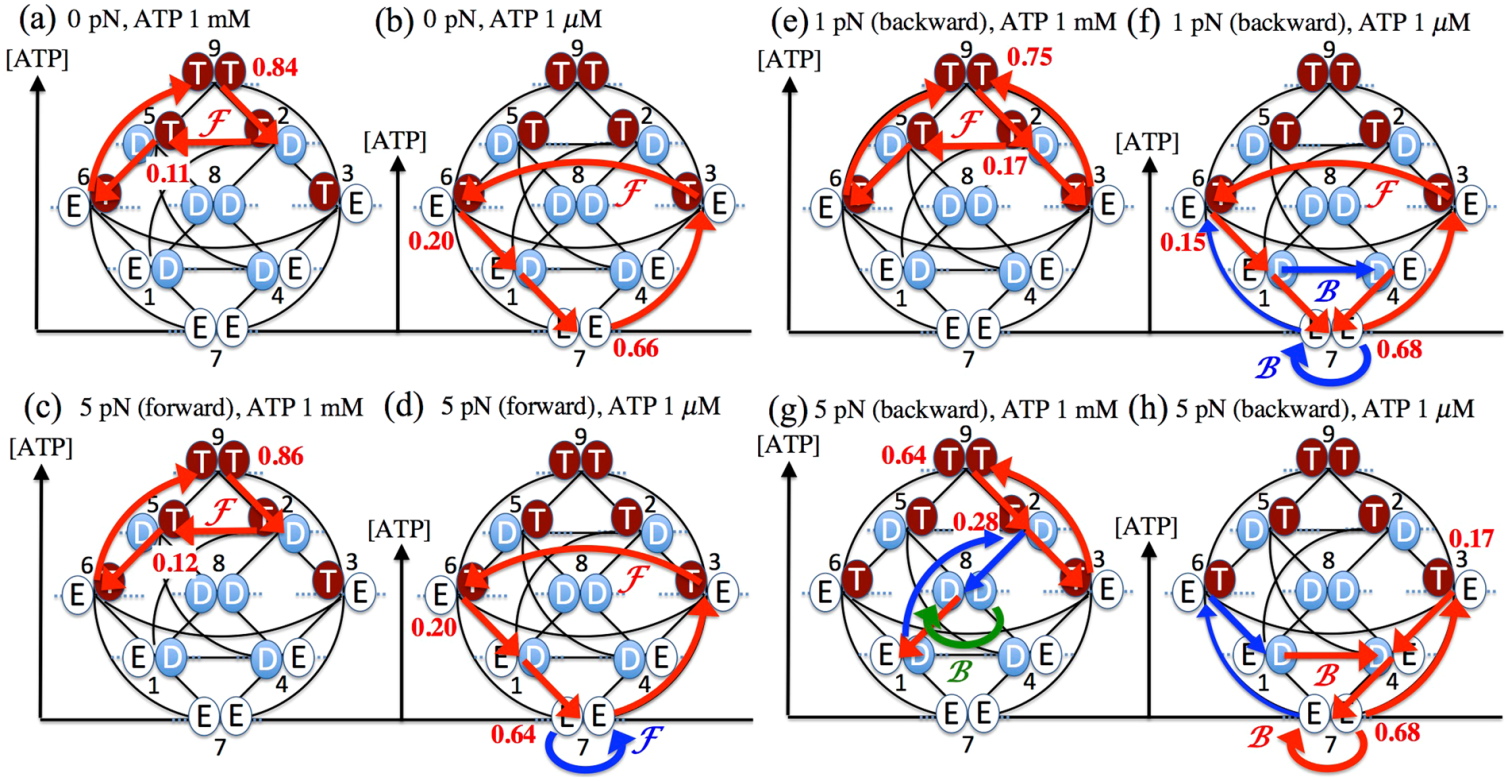
Diagrams of the main local fluxes for high (1 mM) and low (1 µM) ATP concentrations at zero load [(**a**) and (**b**)], a forward-assisting load of 5 pN [(**c**) and (**d**)] and backward loads of 1 pN [(**e**) and (**f**)] and 5 pN [(**g**) and (**h**)], respectively. The red arrows indicate normalized local fluxes larger than 0.10, while the blue arrows indicate other normalized local fluxes larger than 0.07 and the green arrow in Fig. 5g indicates a normalized local flux of 0.03, where the normalized local flux is defined as the value of local flux divided by the sum of all the local fluxes. The red and blue letters  and  indicate the forward- and backward-stepping transitions, respectively. The red numbers indicate the probability of the state. The perpendicular axis for each diagram indicates the ATP-concentration level; higher states on this axis have higher probabilities at high ATP concentrations.

One of the most crucial results obtained in this study are local fluxes on the network at various ATP concentrations and external loads. The nine-state model based on the full-network representation can provide a unified description on the chemomechanical-transition pathways of the working cycle under all chemical and mechanical conditions. Figure 5 shows diagrams of the main local fluxes for high (1 mM) and low (1 µM) ATP concentrations under zero loading [Fig. 5a,b], a forward-assisting load of 5 pN [Fig. 5c,d], and backward loads of 1 pN [Fig. 5e,f] and 5 pN [Fig. 5g,h], respectively. The red arrows indicate normalized local fluxes larger than 0.10, while the blue arrows indicate other normalized fluxes larger than 0.07 and the green arrow in Fig. 5g indicates a normalized flux with 0.03, where these normalized local fluxes are defined in the steady state (st) as the value of the local flux ${J}_{ij}^{{\rm{st}}}={P}_{i}^{{\rm{st}}}{\omega }_{ij}$ [see Method section] divided by the sum of all the local fluxes. The perpendicular axis for each diagram indicates the ATP-concentration level; thus states located at higher places on this axis would be more probable at high ATP concentrations. The red and blue letters *F* and *B* indicate the forward- and backward-stepping transitions, respectively. Although the direction of the mechanical-step transitions from state 2 to state 5, from state 3 to state 6, from state 4 to state 1 is shown to be pointing towards the opposite direction in comparison with Fig. 1a, it is noted that these transitions indicate the forward-stepping transitions.

In Fig. 5a,b, we find ATP-concentration-dependent forward-stepping cycles under the load-free condition. At the high ATP concentration of 1 mM, the main forward cycle |25692> shown in Fig. 5a goes through state 9 (TT), wherein both heads are occupied by ATP; the Pi release from the leading head with a pre-power-stroke conformation, which will result in stabilization of a post-power-stroke conformation for the leading head [see Fig. S4 in the Supplementary Information]^31,40^, causes the force-generating forward-stepping transition from state 2 (TD) to state 5 (DT). This main forward cycle |25692> is completely different from a forward cycle |12581> that has been discussed in previous studies^6,14,22^. If the cycle |12581> appeared as the main forward cycle, ATP hydrolysis or Pi release in the leading head (the 5-to-8 transition) should preferentially take place before the ADP release from the trailing head (the 5-to-6 transition); thus, the rate-limiting transition on the cycle |12581> would be attributed to the ADP release from the trailing head, i.e. the 8-to-1 transition^6,22^, on the basis of the similarity relation between the 8-to-1 and 5-to-6 transitions. As a result, the probability of state 8 (DD) would become high. Since the high probability of state 8 (DD) would cause symmetric random walk even under the load-free condition, it would be hard in the cycle |12581> to provide such high ratio of forward steps to backward steps as shown in Fig. 1c. In previous studies that have accounted for branched kinetic pathways^18,23^, the main forward cycle |25692>, which is provided at the high ATP level by the nine-state model, has been pointed out. Moreover, the necessity of the increase in the probability of state 9 (TT) with increasing ATP concentration for describing the decrease in the mean run length has been discussed in the study as well^18,23^.

In contrast, the main forward cycle |17361> that is obtained by the nine-state model at the low ATP concentration of 1 µM [Fig. 5b] goes through state 7 (EE), wherein both heads are the empty (E) state; it is because ATP binding is the rate-limiting process and the probability of state 7 (EE) becomes highest as the ATP level decreases. It is remarkable that this cycle |17361> includes the 3-to-6 mechanical transition, since this transition has been newly introduced into the network model as a force-generating mechanical-step transition on the basis of the experimental observation by Kodera *et al*. using the AFM technique^30^. The 3-to-6 mechanical transition enables the nine-state model to reproduce the high forward velocity [Figs 1b, 2a,b] and the high chemomechanical-coupling efficiency on converting the hydrolysis of one ATP molecule into one forward step [Fig. 4a] even at low ATP concentrations.

In Fig. 5c, we find that the main forward cycle at the high ATP concentration of 1 mM is the same as that without the assisting load shown in Fig. 5a. The comparison between Fig. 5d,b shows that the load-assisted main-forward cycle at the low ATP concentration of 1 µM is almost the same as that without the assisting load, except that the 7-to-7 forward-stepping transition indicated by the blue arrow in Fig. 5d, which does not directly couple with ATP hydrolysis, is caused by the assisting load. The slight increase in forward velocity at the low ATP concentration of 1 µM with increasing assisting load (as seen in Fig. 1b) and the large effect of assisting load upon the forward velocities at low ATP concentrations (as shown in Fig. 2a,b) are attributable to the load-induced 7-to-7 forward-stepping transition. In any case, the ATP-concentration-dependent forward velocities under the assisting load observed in Figs 1b, 2a,b are basically governed by the main forward cycles driven by ATP hydrolysis indicated as the red arrows in Fig. 5c,d.

In Fig. 5g,h, we find that the backward-stepping cycle is completely different from the reversal of the forward-stepping cycle seen in Fig. 5a,b. This observation indicates that the transduction processes of the free energy by ATP hydrolysis to the mechanical work are irreversible nonequilibrium processes, even if each chemical and mechanical transition on the network is reversible. The irreversibility of the chemomechanical cycles should be attributed to the branched pathways given by the network representation. At the high ATP concentration of 1 mM [Fig. 5g], the main local flux is a futile hydrolysis cycle, |2392>, located on the upper-right-hand side of the network, while the main backward-stepping cycle is due to the 8-to-8 mechanical-slip transition. At the low ATP concentration of 1 µM [Fig. 5h], we find both the 7-to-7 backward mechanical-slip transition and a futile hydrolysis cycle |3473> on the bottom-right-hand side as the main local fluxes. These futile hydrolysis cycles observed at both the ATP concentrations basically go through states at which at least the leading head is strongly bound to the AF, except for state 9 (TT). The results imply that the strong binding to the AF of the leading head inhibits the detachment of myosin V from the AF during load-induced backward stepping. It is noted that these futile hydrolysis cycles would be observed even without accounting for the 8-to-8 and 7-to-7 transitions, since these mechanical-slip transitions have no effect upon the steady-state local fluxes and probabilities. Furthermore, it is hard to describe the sufficiently high velocity of backward stepping under superstall loading using only the reversed 2-to-5 and 3-to-6 transitions, i.e. without accounting for the mechanical-slip transitions. These arguments and the results shown in Figs 5g and 5h indicate that the 8-to-8 and 7-to-7 mechanical-slip transitions and 1-to-4 load-induced mechanical transitions play a crucial role in the high-speed backward-stepping motility of myosin V.

Figure 5,f show that the parts of the local fluxes observed under both zero load [Fig. 5,b] and 5-pN-backward load [Fig. 5e,f] at each ATP concentration appear as the main local fluxes at the backward load of 1 pN.

## Discussion

In an ATPase cycle of myosin^41^, an empty head with the post-power-stroke conformation strongly binds to an AF, and then the ATP binding to the head with this conformation (i.e. the pre-recovery-stroke head) induces detachment of the head from the AF. This observation indicates that the ATP-binding head with the pre-recovery stroke conformation, which corresponds to the trailing head of myosin V, is weakly bound to the AF. Furthermore, it has been demonstrated by Shiroguchi *et al*. that, under the AF-free condition, the post-recovery-stroke conformation is stabilized about 5*k*_B_ *T* by the ATP binding to the head with the pre-recovery-stroke conformation^31^. Thus, the ATP binding to the trailing head with the pre-recovery stroke conformation causes not only the detachment of the head from the AF, but also the recovery stroke simultaneously [see Fig. S4 in the Supplementary Information]. Shiroguchi *et al*. have also pointed out that the post-recovery-stroke conformation can contribute to a preferential binding of the detached head to the forward site on the AF^31^. In the preferential binding to the forward site on the AF by a Brownian search, moderately strong binding to the AF of the ATP-binding or hydrolyzed ADP.Pi-binding head having the post-recovery-stroke conformation would be necessary to suppress the unbinding of this post-recovery-stroke head from the forward site. In fact, the binding ability to the AF of the post-recovery-stroke ADP.Pi head has been experimentally supported by the possibility of a pre-power-stroke closed-cleft conformation that strongly binds to the AF^42^. These experimental observations are consistent with the main forward-stepping cycle provided by the nine-state model at the high ATP concentrations shown in Fig. 5a. The leading heads at states 9 (TT), 5 (DT) and 6 (ET), which appear on the main forward-stepping cycle, correspond to the ATP-binding or ADP.Pi-binding leading head with the pre-power-stroke (post-recovery-stroke) conformation. The binding affinity to the AF of the ATP-binding or ADP.Pi-binding leading head must be moderately strong to achieve the robust motility of myosin V, especially against backward loading.

The more stabilization of the post-recovery-stroke configuration of the ATP-binding or ADP.Pi-binding head than the pre-recovery-stroke conformation^31^ also implies that the latter, i.e. the post-power-stroke conformation is more stabilized than the pre-power-stroke conformation, in other nucleotide states, ADP and empty states [see Fig. S4 in the Supplementary Information]. Thus, the Pi release from the leading head, i.e. the 9-to-2 transition, would stabilize the post-power-stroke conformation of the leading head, thus inducing the force-generating forward step, i.e. the 2-to-5 transition [see Fig. 5a]. This observation suggests a front-head-gating mechanism in the forward-working cycle at the high ATP-concentration levels. On the other hand, at the low ATP concentration, the forward-working cycle goes through state 7 (EE) [see Fig. 5b]. The ATP binding to the trailing head with the pre-recovery-stroke conformation, i.e. the 7-to-3 transition, induces the detachment of the trailing head, and thus the nucleotides-free leading head with the pre-power-stroke conformation in state 3 (TE) (wherein the post-power-stroke conformation has already been more stabilized) causes the force-generating mechanical transition from states 3 (TE) to 6 (ET). This observation is consistent with the single-molecule experiment by Kodera *et al*.; myosin V in the two-head-bound state in the absence of nucleotides as well as in the presence of ADP causes a forward step when the trailing head is unbound from the AF using the AFM technique^30^. These results imply that the forward-working cycle at low ATP concentration levels is interpreted by a rear-head-gating mechanism, where the elastic force between two heads is released when the trailing head is detached from the AF with ATP binding to the head.

## Summary

In this study, we presented a systematic modelling of molecular-motor myosin V on the basis of a chemomechanical network theory^22,24^. A nine-state-network model introduced in this study is based on a full-network representation for two-headed molecular motors with one catalytic domain per head, where one of three different chemical states, i.e. ATP-binding, ADP-binding and empty states, are assigned to each head. In this nine-state model [Fig. 1a], we took into account five different mechanical transitions: between 2 (TD) and 5 (DT), between 3 (TE) and 6 (ET), between 1 (ED) and 4 (DE), between 8 (DD) and 8 (DD) and between 7 (EE) and 7 (EE). Here, the first two transitions produce a forward-biased mechanical step, since the leading head is strongly bound to an actin filament (AF) and the trailing head is easily unbound from the AF, while the last three transitions mainly cause passive mechanical steps when external loading is applied, since both heads are strongly bound to the AF.

We summarize the characteristics of myosin V’s motilities as extracted from the nine-state model in comparison with an eight-state model of kinesin presented in our previous study^28^. In these molecular motors, as a necessary condition for a force-generating mechanical step, at least one head should strongly bind to a cytoskeleton and the other head should be easily unbound from the cytoskeleton. In the case of myosin V, if the leading head with the pre-power-stroke conformation is either ADP-binding or empty state, it will cause the power stroke of the lever arm since the post-power-stroke conformation is more stabilized than the pre-power-stroke conformation^31,40^; thus, myosin V causes the force-generating mechanical step when the trailing head is unbound from the AF [see states 2 (TD) and 3 (TE) in Fig. 1a, which are hereafter referred to as a pre-power-stroke ‘key’ chemical state]. In the case of kinesin, the leading head with an ATP binding causes a movement of the neck linker towards the positive end of a microtubule^43,44^, such that kinesin causes a force-generating mechanical step when the trailing head is unbound from the microtubule [see state 2 (DT) in Fig. 1c of ref.^28^, which is hereafter referred to as a pre-power-stroke ‘key’ chemical state of kinesin]. These two-headed molecular motors commonly use ATP hydrolysis to return a post-power-stroke chemical state (e.g. states 5 (DT) and 6 (ET) for myosin V and state 5 (TD) in Fig. 1c of ref.^28^ for kinesin) to the pre-power-stroke ‘key’ chemical state that causes the force-generating mechanical step. However, it is not obvious which chemical-transition pathways are used by these molecular motors to return the post-power-stroke chemical state back to the pre-power-stroke ‘key’ chemical state. The nine-state model for myosin V without introducing intuitive pre-selection of the chemical and mechanical transitions clearly shows the following things: the local fluxes are not spread over whole the network, but rather are integrated into a main excess flux, while the main cycle or excess flux strongly depends upon both ATP concentration and external load. It is remarkable that (i) the ATP-concentration-dependent main working cycle and (ii) the response of the local excess fluxes to external loading, which are commonly observed for both myosin V and kinesin, are obtained as a pure result of asymmetries and similarity relations between the chemical-transition rates, without introducing any artificial restrictions on transition pathways. It is because, the molecular origin of the asymmetries and similarity relations newly introduced for myosin V in this study is basically interpreted by the intramolecular strain of the leading and trailing heads, in other words, the difference in the catalytic-domain conformations between of the leading and trailing heads with the post-recovery-stroke and pre-recovery-stroke conformations, respectively.

The probabilities of the state at which one head or two heads are occupied by ATP generally increase along with ATP concentration, while the probabilities of the state at which one head or two heads are occupied by no ATPs increases with a decrease in ATP concentration [Fig. 5a,b]. These ATP-dependent state probabilities are not only related to the ATP-concentration dependence of the main working cycle mentioned above in (i), but also lead to qualitatively different ATP-concentration dependences of the mean run lengths for myosin V and kinesin: the mean run length of myosin V is somewhat decreased by an increase in ATP concentration, although the motor velocity is increased [Fig. 3a]; on the other hand, the mean run length of kinesin is increased according to an increase in the motor velocity with increasing ATP concentration. This abnormal decrease in the mean run length of myosin V is attributable to an increase in the detachment rate of myosin V from the AF [Fig. 3c], because the probability of having at least one ATP-binding head (whose binding affinity to the AF is weaker than the other nucleotide states) is increased by the increase in ATP concentration.

As for the response of local fluxes to backward loading mentioned above in (ii), we find that myosin V and kinesin, under superstall loading, cause the main local fluxes to go through the states at which the leading head strongly binds to the cytoskeleton, so that the local fluxes basically works to suppress the unbinding from the cytoskeleton as well as frequent backward stepping. In the case of kinesin, the backward velocity is hardly increased by an increase in backward loading. On the other hand, in the case of myosin V, the velocity is easily increased by the increase in the backward loading [Figs 1b, 2a,b], and the processive high-speed backward motility along the AF is caused by the load-induced mechanical-slip transitions, i.e. the 8-to-8 and 7-to-7 transitions and the load-induced nucleotide-dependent 1-to-4 transition [Fig. 5g,h], wherein both the heads strongly bind to the AF. These observations indicate that myosin V acts as the chemomecanical-coupling ratchet with the load-induced mechanical slip.

## Methods

### Motor dynamics

The dynamics of a molecular motor can be described by a continuous-time Markov process. Thus, the probability ${P}_{i}(t)$ of finding the molecular motor in state *i* at time *t* is governed by the following continuous-time master equations:

1 $$\frac{d}{dt}{P}_{i}(t)=-\sum _{j}\Delta {J}_{ij}(t),$$

2 $$\Delta {J}_{ij}(t)={J}_{ij}(t)-{J}_{ji}(t)={P}_{i}(t){\omega }_{ij}-{P}_{j}(t){\omega }_{ji},$$

where *ω*_*ij*_ is a transition rate from state *i* to state *j*, i.e. the number of transitions from *i* to *j* per unit time, *J*_*ij*_(*t*) and *ΔJ*_*ij*_(*t*); the local flux and local excess flux due to the transition from state *i* to state *j*. In general, the transition rate, *ω*_*ij*_, depends on both the external load parallel to the actin filament, *F*, and the molar concentrations [*X*], where *X* denotes the molecular species ATP, ADP, or Pi (inorganic phosphate). Thus the transition rates can be given by

3 $${\omega }_{ij}={\omega }_{ij}^{0}{\Phi }_{ij}(F),$$

where ${\omega }_{ij}^{0}$ is a zero-force transition rate and ${\Phi }_{ij}(F)$ is a force-dependent factor with ${\Phi }_{ij}(F=0)=1$. Moreover, ${\omega }_{ij}^{0}$ for the binding of an *X*-molecule depends on the molar concentration [*X*] as

4 $${\omega }_{ij}^{0}=\{\begin{array}{c}{\hat{k}}_{ij}[X]\,\\ {k}_{ij}\end{array}\begin{array}{c}{\rm{for}}\,X-\mathrm{binding}\\ {\rm{for}}\,X-\mathrm{release}\end{array},$$

Here, ${\hat{k}}_{ij}$ has dimensions of $1/(\mu {\rm{Ms}})$, while ${k}_{ij}$ has dimensions of 1/s. In the present study, we assume that only the mechanical-transition rates depend upon the external force and that ${\Phi }_{ij}(F)$ for the forward-step transitions under a backward force has the following form (note that the backward force *F* is taken to be positive):

5 $${\Phi }_{ij}(F)=\exp [-F{\theta }_{ij}/{F}_{ij}],$$

6 $${\Phi }_{ji}(F)=\exp [F(1-{\theta }_{ij})/{F}_{ij}],$$

where *F*_*ij*_ is a force scale with respect to the force dependence of the mechanical transition and ${\theta }_{ij}$ is a load-distribution factor with a value of $0 < {\theta }_{ij} < 1$.

### Calculation details

In the nine-state model, the motor velocity *v* is given by $v={v}_{25}+{v}_{36}+{v}_{41}+{v}_{77}+{v}_{88}$, where ${v}_{25}=l\Delta {J}_{25}^{{\rm{st}}}$, ${v}_{36}=l\Delta {J}_{36}^{{\rm{st}}}$, ${v}_{41}=l\Delta {J}_{41}^{{\rm{st}}}$, ${v}_{77}=l{P}_{7}^{{\rm{st}}}({\omega }_{77}^{f}-{\omega }_{77}^{b})$ and ${v}_{88}=l{P}_{8}^{{\rm{st}}}({\omega }_{88}^{f}-{\omega }_{88}^{b})$, with $\Delta {J}_{ij}^{{\rm{st}}}$ and ${P}_{i}^{{\rm{st}}}$ being the local excess flux and the state probability at the steady state, respectively, and ${\omega }_{ii}^{f}$ and ${\omega }_{ii}^{b}$ being the transition rates towards the forward and backward directions, respectively. Here, ${\omega }_{ii}^{f}$ and ${\omega }_{ii}^{b}$ are given by ${\omega }_{ii}^{f}={\omega }_{ii}^{0}\exp [-F{\theta }_{ii}/{F}_{ii}]$ and ${\omega }_{ii}^{b}={\omega }_{ii}^{0}\exp [F(1-{\theta }_{ii})/{F}_{ii}]$, respectively. There are 42 transitions among the nine states other than the 7-to-7 and 8-to-8 mechanical transitions. Using an extension of the steady state balance condition (provided in the Supplementary Information of this work and of ref.^28^) and the similarity relations among the chemical transitions, the number of unknown transition rates can be reduced from 42 to 20 (see the Supplementary Information). The determination of unknown parameters for the transition rates was manually performed as follows. First, we applied the nine-state model [Fig. 1a] to the experimental data on the external load dependence of the motor velocity [Fig. 1b] at several ATP concentrations and the step ratio [Fig. 1c]. Then, we examined the validity of the manually determined parameters by applying to both ATP-concentration dependences of the motor velocity under constant loads [Fig. 2a,b] and ATP- and ADP-concentration dependences of motor velocity without external loading [Fig. 3a,d]. If the theoretical results did not agree well enough with all of the experimental data considered in the present study, this procedure was repeated to refine the parameters until sufficient agreement was achieved. After we determined the parameters for the chemical and mechanical transitions, we applied an extended nine-state model, wherein unbinding transitions of myosin V from the AF via states 9 (TT), 5 (DT), 2 (TD), 6 (ET), 8 (DD), 1 (ED) and 7 (EE) were taken into account [see Fig. 1a and the Supplementary Information], for fitting to the experimental data on the mean run length [Fig. 3b,e] and the detachment rate [Fig. 3c,f] as well. As a final procedure, we recalculated all of the motor properties and refined all the parameters again. It was found that the detachment transitions had only a small effect upon the motor velocity.

## Electronic supplementary material

Supplementary Information
