# Supplementary material for: Myosin V: Chemomechanical-coupling ratchet with load-induced mechanical slip
Source: Sci Rep. 2017 Oct 18;7:13489. doi: 10.1038/s41598-017-13661-0 (PMC5647391; doi:10.1038/s41598-017-13661-0)
Supplement: Supplementary file 1 — Supplementary Information [file 41598_2017_13661_MOESM1_ESM.pdf]

Supplementary Information

Myosin V: Chemomechanical-coupling ratchet with load-induced mechanical slip

Tomonari Sumi[1,2]

[1]Research Institute for Interdisciplinary Science and [2]Department of Chemistry, Faculty of Science, Okayama University, 3-1-1 Tsushima-Naka, Kita-ku, Okayama 700-8530, Japan

An extension of the steady state balance condition

The steady state balance condition has been presented by Liepelt and Lipowsky [1] as generalization of the detailed balance condition in equilibrium which would provide the necessary condition in nonequilibrium steady state for nanomachine. In their formulation, the first law of thermodynamics is applied to individual cycles and the second law with the equality for reversible process is used to relate the statistical entropy produced by the individual cycle to the heat released from the system. However, in my understanding, we have to apply the second law of thermodynamics based on both the equality and inequality to characterize irreversible cycles of nanomachine in nonequilibrium steady state[2]. According to the formulation presented by Liepelt and Lipowsky[1], let's start with the conservation of energy during the completion of individual cycle. A change in the internal energy $\Delta U\left(C_\nu^+\right)$ along any directed cycles C_ν^+ satisfies

$$\Delta U\left(C_\nu^+\right) = E_{chem}\left(C_\nu^+\right) - W_{mech}\left(C_\nu^+\right) - Q\left(C_\nu^+\right), \tag{S1}$$

where $E_{chem}\left(C_\nu^+\right)$ is an energy input given by chemical reactions, $W_{mech}\left(C_\nu^+\right)$; a mechanical work performed by mechanical transitions against an external load, and $Q\left(C_\nu^+\right)$; heat released from the system, during the completion of the directed cycle C_ν^+.

In this study, we assume that the motor dynamics is described by a continuous-time Markov process with transition rates ω_{ij} from state *i* to state *j*. The statistical entropies $\Delta S\left(C_\nu^+\right)$ and $\Delta S\left(C_\nu^-\right)$, which are produced in the steady state during the completion of directed cycles C_ν^+ and C_ν^-, are provided by[1,3]

$$\Delta S\left(C_\nu^+\right) = k_B \sum_{|ij\rangle}^{\nu,+} \ln\left(\omega_{ij}/\omega_{ji}\right) \equiv -\Delta S\left(C_\nu^-\right), \tag{S2}$$

where the summation is performed over all edges $|ij\rangle$ along the directed cycle C_ν^+. Now we apply the second law of thermodynamics to relate the statistical entropy $\Delta S\left(C_\nu^+\right)$ to the heat released from the

system:

$$\varDelta S\left(C_\nu^+\right) \geq Q\left(C_\nu^+\right)\big/T\,, \tag{S3}$$

where the equality holds if the directed cycle C_ν^+ is reversible. Using Eqs. (S1)-(S3), we obtain the following equation:

$$\left\{E_{chem}\left(C_\nu^+\right) - W_{mech}\left(C_\nu^+\right)\right\}\big/k_B T - \sum\nolimits_{|ij\rangle}^{\nu,+} \ln\left(\omega_{ij}/\omega_{ji}\right) \leq 0\,, \tag{S4}$$

which can be regarded as an extended steady state balance condition, where only the inequality in Eq. (S4) is different from that originally presented by Liepelt and Lipowsky[1]. The irreversibility of the directed cycles resulting in the inequality of Eq. (S4) should be attributable to the non-zero excess flux $\varDelta J_\nu^+$ for the directed cycle C_ν^+ or the non-zero $\varDelta J_\nu^+$ for its reversed cycle C_ν^-. In the case of molecular motor myosin V, the large difference between the main forward-stepping cycle and the main backward-stepping cycle seems to reflect the irreversibility of the transduction process of ATP-hydrolysis free energy into mechanical work.

Here the chemical free-energy input per directed cycle can be expressed as

$$E_{chem}\left(C_\nu^d\right) = \left[n_{\mathrm{h}}\left(C_\nu^d\right) - n_{\mathrm{s}}\left(C_\nu^d\right)\right]\varDelta\mu\,, \tag{S5}$$

$$\Delta\mu = k_B T \ln\left\{\left(K_{eq}[\mathrm{ATP}]\right)\big/\left([\mathrm{ADP}][\mathrm{P}]\right)\right\}, \tag{S6}$$

where $n_{\mathrm{h}}\left(C_\nu^d\right)$ and $n_{\mathrm{s}}\left(C_\nu^d\right)$ are respectively the numbers of hydrolysis and synthesis that are contained in one directed cycle of C_ν^d, and $\Delta\mu$ is the excess free energy change that depends on the concentrations of [ATP], [ADP], and [P], and is given by one ATP hydrolysis, resulting in binding one ATP to the motor and releasing one ADP and one P from the motor, where the standard state for the change in $\Delta\mu$ is the chemical equilibrium with the equilibrium constant K_{eq} [4,5]. In the presence of an external load *F*, the mechanical work performed by the molecular motor during the completion of the directed cycle C_ν^d is given by

$$W_{mech}\left(C_\nu^d\right) = \left[m_{\mathrm{f}}\left(C_\nu^d\right) - m_{\mathrm{b}}\left(C_\nu^d\right)\right] lF\,, \tag{S7}$$

where *l* is the size of the mechanical step and $m_{\mathrm{f}}\left(C_\nu^d\right)$ and $m_{\mathrm{b}}\left(C_\nu^d\right)$ are respectively the numbers of the mechanical forward and backward steps that are contained in one directed cycle of C_ν^d. Substituting Eqs. (S5) and (S7) into Eq. (S4), we obtain

$$\left\{\left[n_{\mathrm{h}}\left(C_\nu^+\right) - n_{\mathrm{s}}\left(C_\nu^+\right)\right]\varDelta\mu - \left[n_{\mathrm{f}}\left(C_\nu^+\right) - n_{\mathrm{b}}\left(C_\nu^+\right)\right] Fl\right\}\big/k_B T - \sum\nolimits_{|ij\rangle}^{\nu,+} \ln\left(\omega_{ij}/\omega_{ji}\right) \leq 0\,. \tag{S8}$$

Now we assume that the transition rates are given by Eqs. (3)-(6). To extract any information from Eq. (S8), we consider the case of F=0 where all of the force dependent factors $\Phi_{ij}(F)$ vanish and thus the following equation is obtained:

$$\left[n_{\mathrm{f}}\left(C_v^+\right)-n_{\mathrm{b}}\left(C_v^+\right)\right]\Delta\mu/k_{\mathrm{B}}T-\sum_{|ij\rangle}^{v,+}\ln\left(\omega_{ij}^0/\omega_{ji}^0\right)\leq 0\,. \tag{S9}$$

If the excess fluxes $\Delta J_v^+ s$ for all the directed cycles are equal to zero, the system is in thermodynamic equilibrium, thus all the directed cycles are reversible and then the equality in Eq. (S9) should hold. As a result, we obtain

$$\sum_{|ij\rangle}^{v,+}\ln\left(\omega_{ij}^0/\omega_{ji}^0\right)=\left[n_{\mathrm{f}}\left(C_v^+\right)-n_{\mathrm{b}}\left(C_v^+\right)\right]\Delta\mu/k_{\mathrm{B}}T=0\,, \tag{S10}$$

which corresponds to the detailed balance conditions in equilibrium presented by Liepelt and Lipowsky[1]. It has been pointed out by them that the detailed balance conditions in equilibrium are fully satisfied, if Eq. (S10) is applied to all the fundamental cycles.

Next, we substitute Eqs. (3), (4), and (S9) into Eq. (S8), and then we obtain the following condition under external loading:

$$\sum_{|ij\rangle}^{v,+}\ln\left(\Phi_{ij}(F)/\Phi_{ji}(F)\right)\geq-\left[m_{\mathrm{f}}\left(C_v^+\right)-m_{\mathrm{b}}\left(C_v^+\right)\right]Fl/k_{\mathrm{B}}T\,, \tag{S11}$$

where the equality holds in reversible process. In the case that all the directed cycles in the system include the mechanical transitions–a single cycle system is a typical simplest case–at a special stall load condition where $\Delta J_v^+ s$ for all the directed cycles are equal to zero, the system should be in thermal and mechanical equilibrium. In this case, the directed cycles are reversible and thus the equality in Eq. (S11) should hold. As a result, we obtain

$$\sum_{|ij\rangle}^{v,+}\ln\left(\Phi_{ij}(F)/\Phi_{ji}(F)\right)=-\left[m_{\mathrm{f}}\left(C_v^+\right)-m_{\mathrm{b}}\left(C_v^+\right)\right]Fl/k_{\mathrm{B}}T\,, \tag{S12}$$

which is equivalent to a part of the steady state balance condition presented by Liepelt and Lipowsky[1], and gives a relation $1/F_{ij}=l/k_{\mathrm{B}}T$ by substituting Eqs. (5) and (6) into Eq. (S12).

In contrast, in the case that the system contains directed cycles without mechanical transitions, in other words, possible futile chemical cycles, even at the stall load conditions so that $\Delta J_v^+ s$ for the directed cycles including the mechanical transitions could be equal to zero, the futile chemical cycles that contain no mechanical transitions [for instance the cycles such as |2392> and |3473> seen in Figs. 5g and 5h] would be driven by the free-energy input. However, this situation seems to be rather special case at which all of $\Delta J_v^+ s$ for the directed cycles including the mechanical transitions become simultaneously zero under the

stall load condition if the system has a lot of mechanical transitions like myosin V. This is because the stall load condition is basically achieved if the sum of all the velocity components arising from the mechanical transitions becomes zero [for instance, in the nine-state model for myosin V, $v = v_{25} + v_{36} + v_{41} + v_{77} + v_{88}$ should become zero under the stall load condition, whereas each velocity component does not have to necessarily become zero], indicating that ΔJ_v^+ for each directed cycle is not necessary being zero at the stall load condition. In these cases discussed above as possible examples, the system is not in equilibrium and then the directed cycles are irreversible; thus the inequality in Eq. (S11) should hold. As a result, we obtain the following condition:

$$\sum_{|ij\rangle}^{v,+} \ln\left(\Phi_{ij}(F)/\Phi_{ji}(F)\right) > -\left[m_{\mathrm{f}}\left(C_v^+\right) - m_{\mathrm{b}}\left(C_v^+\right)\right] Fl \Big/ k_{\mathrm{B}}T \,. \tag{S13}$$

In our model, Eq. (S13) is satisfied by a condition $1/F_{ij} < l/k_{\mathrm{B}}T$ that is provided by substituting Eqs. (5) and (6) into Eq. (S13). To satisfy the extended steady state balance condition provided by Eq. (S4), we impose the detailed balance conditions in equilibrium, i.e. Eq. (S10), to the zero-force transition rates ω_{ij}^0 given by Eq. (4) and also apply the condition $1/F_{ij} < l/k_{\mathrm{B}}T$ that is provided by Eq. (S13) to the force-dependent factor given by Eqs. (5) and (6).

Theoretical models that have been proposed for myosin V

Bierbaum and Lipowsky[6] proposed a six-state chemomechanical network model for myosin V, where states 3 (TE), 4 (DE), and 9 (TT) were omitted from the nine-state full representation [Fig. 1 (a)] and only the mechanical transitions between states 2 (TD) and 5 (DT) and between states 7 (EE) and 7 (EE) were taken into account. Based on the effect on the chemical-transition rates caused by the difference between post-recovery-stroke and pre-recovery-stroke conformations [see Fig. S4] of the leading and trailing heads, respectively, i.e. the intramolecular strain of the leading and trailing heads, the 1-to-2 transition, i.e. the ATP binding to the trailing head should be similar to the 6-to-9 and 7-to-3 transition; the 1-to-7 transition, i.e. ADP release from the leading head should be similar to the 2-to-3 and 8-to-4 transitions. The 7-to-7 mechanical transition, wherein both heads are strongly bound to an actin filament, would be similar to the 8-to-8 mechanical transition. Thus, these chemical and mechanical transitions should be taken into account to resolve the internal inconsistencies among the transition rates that arise by omitting them as well.

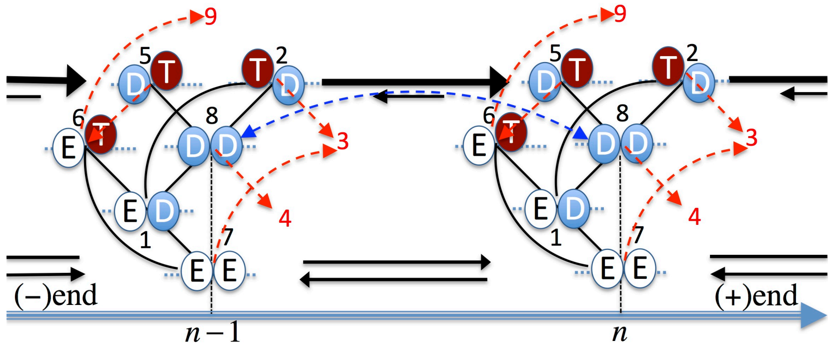

Figure S1. A six-state model for myosin V proposed by Bierbaum and Lipowsky [6]. Black solid and black solid directed lines indicate the chemical and mechanical transitions, respectively. Red and blue broken directed lines respectively show the chemical and mechanical transitions that should be taken into account to resolve the internal inconsistencies among the transition rates; the similarity relations (concerned with the 5-to-6, 6-to-9, 2-to-3, 8-to-4, and 7-to-3 transitions) between chemical transitions are suggested by the intramolecular strain and a similarity relation (concerned with the 8-to-8 transition) between mechanical transitions is suggested by the binding affinity of heads to the actin filament.

Zhange *et al.*[7] proposed a six-state chemomechanical network model for myosin V, where states 3 (TE), 4 (DE), and 7 (EE) were omitted from the nine-state full representation [Fig. 1 (a)] and only the mechanical transitions between states 2 (TD) and 5 (DT) was taken into account. Basically, no internal inconsistencies as seen in the six-state model by Bierbaum and Lipowsky[6] are found in the network structure. However, we find a few problems that are expected to occur at either low ATP concentrations or under high backward loading: the 2-to-3 transition would take place if a high backward load is applied; the 1-to-7 transition would occur at low ATP concentrations. Note that these omitted transitions are the ADP release from the leading head. The need for these chemical transitions would be verified by applying the model including these transitions to the experimental data. Furthermore, we notice an internal inconsistency between chemical-transition rates obtained as ATP hydrolysis on the leading head, i.e. the 5-to-8 and 6-to-1 transitions. In their six-state model, it is assumed as follows: ATP hydrolysis on the leading head would preferentially take place, while that on the trailing head, e.g. the 9-to-5 and 2-to-8 transitions hardly occur so that these chemical transitions are omitted from the network. This assumption is justified by considering the effect of the intramolecular strain on the chemical transitions. However, this effect should simultaneously lead the similarity relation between the 5-to-8 and 6-to-1 transitions, because the leading head are strongly pulled backward by the trailing head that is strongly bound to the AF, during these ATP-hydrolysis reactions on the leading head. However, the rate determined for the 5-to-8 transition (6 s^{-1}) is significantly different from that for the 6-to-1 transition (294 s^{-1}). Not only the asymmetries but also the similarity relations between the chemical transitions should be simultaneously satisfied to resolve this internal inconsistency.

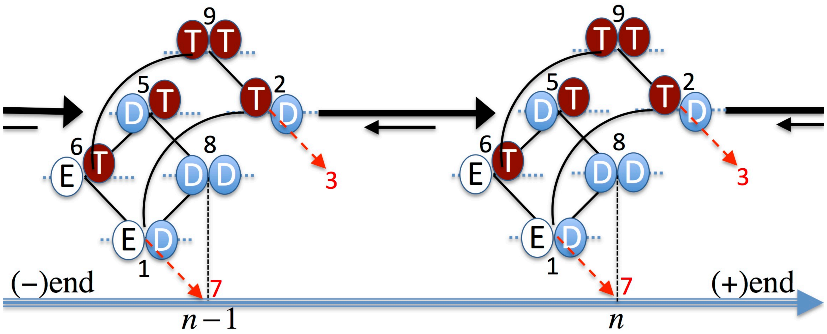

Figure S2. A six-state model for myosin V proposed by Zhang *et al.*[7] Black solid lines and black directed lines indicate chemical and mechanical transitions, respectively. Red directed broken lines show the transitions that should be taken into account to resolve the problems concerned with the 2-to-3 and 1-to-7 transitions, which would arise under high backward loading and at low ATP concentrations, respectively.

The detailed balance conditions for the nine-state model

In the graph theory, a fundamental cycle basis of an undirected graph is given by a set of simple cycles that forms a basis of the cycle space of the graph. The number of fundamental cycles N_{fc} in a given connected graph is provided as $N_e - N_v + 1$ where N_e is the number of edges and N_v is the number of vertices [8]. In the case of nine-state chemomechanical network model [Fig. 1 (a)], the number of the fundamental cycles is obtained as $N_{fc} = 13$ because of $N_e=21$ and $N_v=9$. Therefore, thirteen independent equations among the transition rates are provided by the detailed balance condition [1]. Thirteen fundamental cycles are displayed in Fig. S1. Each fundamental cycle provides the following equations:

(1) Cycle <234716592>

$$k_{23}k_{34}k_{47}k_{47}\hat{k}_{71}\hat{k}_{71}\hat{k}_{16}\hat{k}_{65}\hat{k}_{59}k_{92} \Big/ \hat{k}_{32}\hat{k}_{29}k_{95}k_{56}k_{61}k_{17}\hat{k}_{74}\hat{k}_{43} = 1 . \quad \text{(S14)}$$

(2) Cycle<2952>

$$\hat{k}_{29}k_{95}\omega_{52}^{0} \Big/ k_{92}\omega_{25}^{0}\hat{k}_{59} = 1 . \quad \text{(S15)}$$

(3) Cycle<2582>

$$k_{28}\hat{k}_{85}\omega_{52}^{0} \Big/ \hat{k}_{82}\omega_{25}^{0}k_{58} = 1 . \quad \text{(S16)}$$

(4) Cycle <18561>

$$\hat{k}_{18}\hat{k}_{85}k_{56}k_{61} \Big/ k_{81}\hat{k}_{16}\hat{k}_{65}k_{58} = 1 . \quad \text{(S17)}$$

(5)Cycle <28432>

$$k_{28}k_{84}\hat{k}_{43}\hat{k}_{32} \Big/ \hat{k}_{82}k_{23}k_{34}\hat{k}_{48} = 1 . \quad \text{(S18)}$$

(6) Cycle <1471>

$$\omega_{14}^{0}k_{47}\hat{k}_{71} \Big/ \omega_{41}^{0}k_{17}\hat{k}_{74} = 1 . \quad \text{(S19)}$$

(7) Cycle<9673>

$$k_{96}k_{67}\hat{k}_{73}\hat{k}_{39} \Big/ \hat{k}_{69}k_{93}k_{37}\hat{k}_{76} = 1 . \quad \text{(S20)}$$

(8)Cycle<2392>

$$k_{23}\hat{k}_{39}k_{92} \Big/ \hat{k}_{32}\hat{k}_{29}k_{93} = K_{eq} . \quad \text{(S21)}$$

(9) Cycle <6176>

$$k_{61}k_{17}\hat{k}_{76} \Big/ \hat{k}_{16}k_{67}\hat{k}_{71} = K_{eq} . \quad \text{(S22)}$$

(10) Cycle <3473>

$$k_{34}k_{47}\hat{k}_{73} \Big/ \hat{k}_{43}k_{37}\hat{k}_{74} = K_{eq} . \quad \text{(S23)}$$

(11) Cycle <174361>

$$k_{17}\hat{k}_{74}\hat{k}_{43}\omega_{36}^{0}k_{61} \Big/ \hat{k}_{71}\hat{k}_{16}\omega_{63}^{0}k_{34}k_{47} = 1 . \quad \text{(S24)}$$

(12) Cycle<2956182>

$$\hat{k}_{29}k_{95}k_{56}k_{61}\hat{k}_{18}\hat{k}_{82} \Big/ k_{92}k_{28}k_{81}\hat{k}_{16}\hat{k}_{65}\hat{k}_{59} = 1 . \quad \text{(S25)}$$

(13) Cycle<5923485>

$$\hat{k}_{59}k_{92}k_{23}k_{34}\hat{k}_{48}\hat{k}_{85} \big/ k_{95}k_{58}k_{84}\hat{k}_{43}\hat{k}_{32}\hat{k}_{29} = 1\,. \qquad \text{(S26)}$$

We can use these thirteen equations to reduce the number of unknown transition rates.

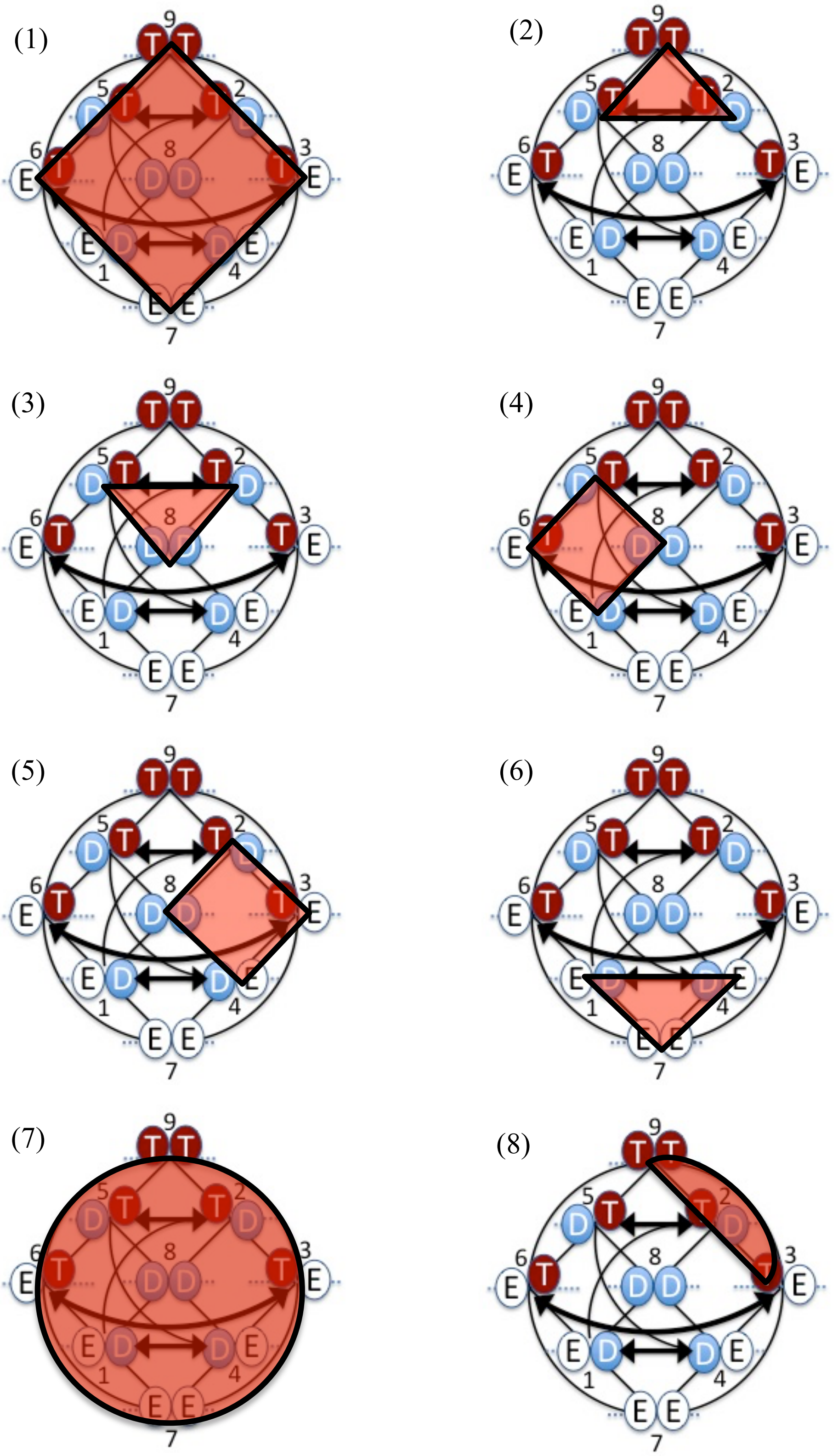

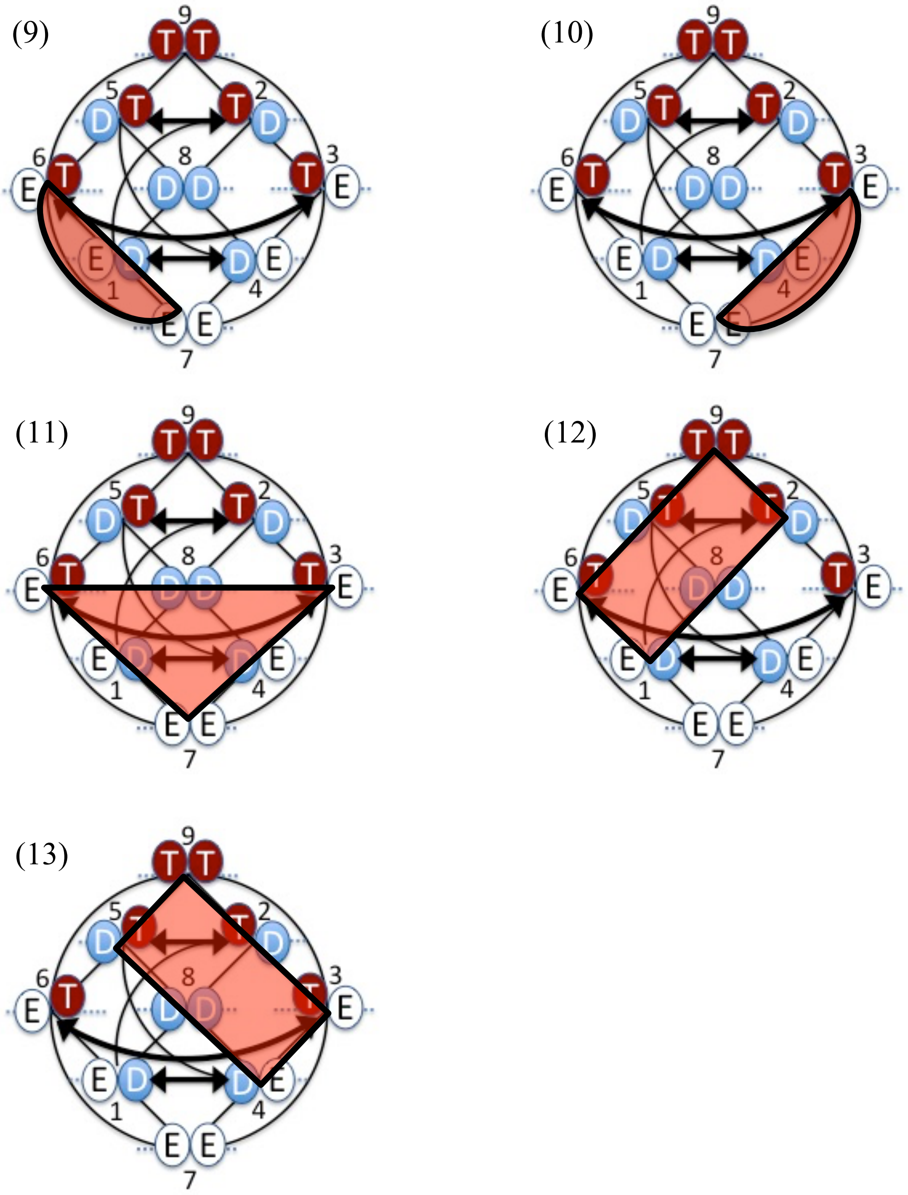

Figure S3. Thirteen fundamental cycles for the nine-state network model. The red mesh in each figure indicates a fundamental cycle.

Asymmetries and similarity relations between transition rates

There are 42 (= 2 N_e) transitions among nine states in the nine-state network model except for the 7-to-7 and 8-to-8 transitions. Based on the effect on the chemical-transition rates caused by the intramolecular strain of the leading and trailing heads, in other words, the difference between the post-recovery-stroke and pre-recovery-stroke conformations [see Fig. S4] for the leading and trailing heads, respectively, we introduce asymmetries and similarity relations between transition rates to reduce the number of unknown transition rates systematically. For example, we here consider the chemical transitions from state 9 (TT) to state 2 (TD) and from state 9 (TT) to state 5 (DT). Although these transitions are ATP-hydrolysis reactions, the 9-to-2 transition is ATP hydrolysis on the leading head that is pulled backward by the partner trailing head, while the 9-to-5 transition is ATP hydrolysis on the trailing head that is pulled forward by the partner leading head. This observation indicates that the rates for these transitions would be affected by the intramolecular strain of the leading and trailing heads and thus would be different each other. This asymmetry between these chemical-transition rates would be attributable to the different conformations of the catalytic domains in the leading and trailing heads with post-recovery and pre-recovery conformations, respectively. [see Fig. S4]. On the other hand, for instance, we can find a similarity relation between the chemical transitions from state 5 (DT) to state 8 (DD) and from state 6 (ET) to state 1 (ED); these transitions are ATP hydrolysis on the leading head with the post-recovery-stroke conformation [see Fig. S4], wherein the leading head is strongly pulled backward by the partner trailing head that is strongly bound to the actin filament (AF). In the same manner, we also find similarity relations between the transitions from state 2 (TD) to state 8 (DD) and from state 3 (TE) to state 4 (DE).

According to the intramolecular strain of the leading and trailing heads depending on the binding affinity of the partner head to the AF, we assume the following asymmetries and similarity relations between transition rates:

(1) ATP hydrolysis/synthesis on the trailing head

$$\omega_{28} = \omega_{34}, \tag{S27a}$$

$$\omega_{82} = \omega_{43}. \tag{S27b}$$

(2) ATP hydrolysis/synthesis on the leading head

$$\omega_{58} = \omega_{61}, \tag{S28a}$$

$$\omega_{85} = \omega_{16}. \tag{S28b}$$

(3) ATP binding to/release from the trailing head

$$\omega_{12} = \omega_{73}, \tag{S29a}$$

$$\omega_{21} = \omega_{37}. \tag{S29b}$$

(4) ATP binding to/release from the leading head

$$\omega_{45} = \omega_{76}, \tag{S30a}$$

$$\omega_{54} = \omega_{67}. \tag{S30b}$$

(5) ADP binding to/release from the trailing head

$$\omega_{18} = \omega_{74}, \tag{S31a}$$

$$\omega_{81} = \omega_{47}. \tag{S31b}$$

(6) ADP binding to/release from the leading head

$$\omega_{48} = \omega_{71}, \tag{S32a}$$

$$\omega_{84} = \omega_{17}. \tag{S32b}$$

These similarity relations between the chemical transitions reduce the number of unknown transition rates from 42 to 30.

If we substitute these 12 relations to Eqs. S14–S26 that are provided by the detailed balance condition, we finally obtain the following ten independent detailed balance conditions:

(2) Cycle <2952>

$$\hat{k}_{59} = \hat{k}_{29} k_{95} \omega_{52}^{0} / k_{92} \omega_{25}^{0}. \tag{S33}$$

(3) Cycle <2582>

$$\hat{k}_{85} = \omega_{25}^{0} k_{58} \hat{k}_{82} / \omega_{52}^{0} k_{28}. \tag{S34}$$

(4) Cycle <18561>

$$\hat{k}_{18} = \hat{k}_{65}\, k_{47} / k_{56}, \tag{S35a}$$

$$\hat{k}_{74} = \hat{k}_{18}. \tag{S35b}$$

(5) Cycle <28432>

$$\hat{k}_{48} = k_{17} \hat{k}_{32} / k_{23}, \tag{S36a}$$

$$\hat{k}_{71} = \hat{k}_{48}. \tag{S36b}$$

(6) Cycle <1471>

$$\omega_{41}^{0} = \omega_{14}^{0} \hat{k}_{32} k_{56} / \hat{k}_{65} k_{23}. \tag{S37}$$

(7) Cycle <9673>

$$k_{96} = \omega_{25}^{0} \hat{k}_{69} k_{92} k_{56} \Big/ \left(\omega_{52}^{0} \hat{k}_{29} \hat{k}_{65} K_{eq} \right). \tag{S38}$$

(8) Cycle <2392>

$$k_{93} = k_{23}\, \hat{k}_{39} k_{92} \Big/ \left(\hat{k}_{32} \hat{k}_{29} K_{eq} \right). \tag{S39}$$

(9) Cycle <6176>

$$k_{54} = \hat{k}_{45} \omega_{52}^{0} k_{23} k_{28} \Big/ \left(\hat{k}_{32} \omega_{25}^{0} \hat{k}_{82} K_{eq} \right). \tag{S40}$$

(10) Cycle <3473>

$$k_{21} = \hat{k}_{12} k_{56} k_{28} \Big/ \left(\hat{k}_{65} \hat{k}_{82} K_{eq} \right). \tag{S41}$$

(11) Cycle <174361>

$$\omega_{63}^{0} = \omega_{36}^{0} \omega_{52}^{0}\, \hat{k}_{65} k_{23} / \omega_{25}^{0} \hat{k}_{32} k_{56}. \tag{S42}$$

Here, K_{eq} is the equilibrium constant,

$$K_{eq} = \left.\frac{[\mathrm{ADP}][\mathrm{P}]}{[\mathrm{ATP}]}\right|_{eq} \simeq 4.9\times 10^{11}\mu\mathrm{M}^{9}, \tag{S43}$$

where $[\mathrm{ATP}]$, $[\mathrm{ADP}]$, and $[\mathrm{P}]$ are the concentrations of ATP, ADP, and Pi (inorganic phosphate), respectively. As a result, the number of unknown transition rates is reduced to 20 from 30.

Here, the transition from state 3 (TE) to state 9 (TT) is the ATP binding to the leading head and this leading head has a pre-power-stroke or post-recovery-stroke conformation [see Fig. S4], whereas the partner trailing head with ATP binding is weakly bound to the AF. During the transition from state 4 to state 5 and from state 7 to state 6, the leading head to which ATP is going to bind also has a pre-power-stroke or post-recovery-stroke conformation, although the partner trailing head is strongly bound to the AF. Based on the pre-power-stroke or post-recovery-stroke conformation of the leading head to which ATP is going to bind, the transition rate from state 3 (TE) to state 9 (TT) would be much more similar to the transition rate from state 4 to state 5 and from state 7 to state 6 than that from state 6 (ET) to 9 (TT); it is because, the trailing head to which ATP binds during the transition from state 6 (ET) to 9 (TT) has a pre-recovery-stroke or post-power-stroke conformation. In the same manner, the transition rate from state 6 (ET) to state 9 (TT) would be much more similar to the transition rate from state 1 to state 2 and from state 7 to state 3 than that from state 3 (TE) to state 9 (TT). Therefore, we can suppose the following approximate relations:

(1) ATP binding to/release from the leading head with a pre-power-stroke or post-recovery-stroke conformation:

$$\omega_{39} \approx \omega_{45} = \omega_{76}, \tag{S44a}$$

$$\omega_{93} \approx \omega_{54} = \omega_{67}. \tag{S44b}$$

(2) ATP binding to/release from the trailing head with a pre-recovery-stroke or post-power-stroke conformation:

$$\omega_{69} \approx \omega_{12} = \omega_{73}, \tag{S45a}$$

$$\omega_{96} \approx \omega_{21} = \omega_{37}. \tag{S45b}$$

In addition, in the same manner, we can suppose the following approximate relations:

(3) ATP hydrolysis/synthesis on the leading head with a pre-power-stroke or post-recovery-stroke conformation:

$$\omega_{92} \approx \omega_{58} = \omega_{61}, \tag{S46a}$$

$$\omega_{29} \approx \omega_{85} = \omega_{16}. \tag{S46b}$$

(4) ATP hydrolysis/synthesis on the trailing head with a pre-recovery-stroke or post-power-stroke conformation:

$$\omega_{95} \approx \omega_{28} = \omega_{34}, \tag{S47a}$$

$$\omega_{59} \approx \omega_{82} = \omega_{43}. \tag{S47b}$$

Detachment of myosin V from an actin filament

It has been experimentally demonstrated that the head in state D or E is strongly bound to an AF while the head in state T is weakly bound to an AF[10]. Therefore, we expect that the detachment of myosin V from an AF would more frequently take place via states at which either one head or both heads are occupied by ATP. This observation implies that the detachment of myosin V from the AF is increased with increasing ATP concentration. In addition, even if both heads are strongly bound to the AF, the detachment via the states strongly binding to the AF would occasionally be observed if the residence time in these states is longer than that in the other states. Based on these considerations, we took into account the detachment transitions from states 9 (TT), 5 (DT), 2 (TD), 6 (ET), 8(EE), 1(ED), and 7(EE) into an unbinding state (absorbing state) "0" under backward loading as follows:

$$\omega_{i0} = k_{i0}\Omega_{i0}\left(F\right) \quad , \tag{S48}$$

where k_{i0} is a zero-force detachment rate and $\Omega_{i0}\left(F\right)$ is a force-dependent factor for the detachment transition under load,

$$\Omega_{i0}\left(F\right) = \exp\left[\left|F\right|/F_{i0}\right]. \tag{S48}$$

Here, F_{i0} is a force scale with respect to the force dependence of the detachment transition via state i under load.

According to the Hill's method for the mean time to absorption[11,12], the mean time of binding or unbinding rate can be calculated using a modified diagram in which each absorption state is replaced by a one-way cycle back to a starting state. In this study, we employed states 8 (DD) as the starting state to determine the mean time of binding.

The transition rates obtained using the global fitting by the nine-state model to available experimental data on myosin V's motor properties

The transition rates obtained using the nine-state model should be regarded as optimal values for describing the motor dynamics in nonequilibrium steady states, and those values could be different from the rate constants that have been determined by biochemical experiments under thermodynamic equilibrium. Nevertheless, in the point of view of the asymmetries and similarity relations between the transition rates on the leading and trailing heads as discussed above, the obtained values would be qualitatively consistent with that determined by biochemical experiments under thermodynamic equilibrium.

Table S1. The chemical transition rates in the nine-state network model.

Parameters	Meaning	Values
k_{56}	ADP release from the trailing head	90.0 s^{-1}
$\hat{k}_{65}$	ADP binding to the trailing head	170 $(\mu Ms)^{-1}$
k_{23}	ADP release from the leading head	30.0 s^{-1}
$\hat{k}_{32}$	ADP binding to the leading head	50.0 $(\mu Ms)^{-1}$
k_{95}	ATP hydrolysis on the trailing head	1.00 s^{-1}
$\hat{k}_{59}$	ATP synthesis on the trailing head*	1.65 x 10^{-7} $(\mu Ms)^{-1}$
k_{92}	ATP hydrolysis on the leading head	11.5 s^{-1}
$\hat{k}_{29}$	ATP synthesis on the leading head	2.62 x 10^{-3} $(\mu Ms)^{-1}$
$\hat{k}_{69}$	ATP binding to the trailing head	1.90 $(\mu Ms)^{-1}$
k_{96}	ATP release from the trailing head*	1.24 x 10^{-5} s^{-1}
$\hat{k}_{39}$	ATP binding to the leading head	0.900 $(\mu Ms)^{-1}$
k_{93}	ATP release from the leading head*	4.85 x 10^{-9} s^{-1}
$k_{58} = k_{61}$	ATP hydrolysis on the leading head	10.0 s^{-1}
$\hat{k}_{85} = \hat{k}_{16}$	ATP synthesis on the leading head*	1.72 x 10^{-3} $(\mu Ms)^{-1}$
$k_{28} = k_{34}$	ATP hydrolysis on the trailing head	8.00 s^{-1}
$\hat{k}_{82} = \hat{k}_{43}$	ATP synthesis on the trailing head	1.00 x 10^{-6} $(\mu Ms)^{-1}$
$k_{17} = k_{84}$	ADP release from the leading head	15.0 s^{-1}
$\hat{k}_{71} = \hat{k}_{48}$	ADP binding to the leading head*	25.0 $(\mu Ms)^{-1}$
$k_{47} = k_{81}$	ADP release from the trailing head	90.0 s^{-1}
$\hat{k}_{74} = \hat{k}_{18}$	ADP binding to the trailing head*	170 $(\mu Ms)^{-1}$
$\hat{k}_{12} = \hat{k}_{73}$	ATP binding to the trailing head	1.90 $(\mu Ms)^{-1}$
$k_{21} = k_{37}$	ATP release from the trailing head*	1.64 x 10^{-5} s^{-1}
$\hat{k}_{45} = \hat{k}_{76}$	ATP binding to the leading head	1.00 $(\mu Ms)^{-1}$
$k_{54} = k_{67}$	ATP release from the leading head*	7.12 x 10^{-9} s^{-1}

[*] Determined by the detailed balance condition.

Table S2. The mechanical-step transition rates in the nine-state network model.

Parameters	Meaning	Values
ω_{25}^{0}	Forward transition from state 2 to state 5	550 s^{-1}
ω_{52}^{0}	Backward transition from state 5 to state 2	0.400 s^{-1}
F_{25}	Force dependence on the 2–5 transition	0.331 pN
θ_{25}	Load distribution between states 2 and 5	0.9999
ω_{36}^{0}	Forward transition from state 3 to state 6	330 s^{-1}
ω_{63}^{0}	Backward transition from state 6 to state 3	0.272 s^{-1}
F_{36}	Force dependence on the 3–6 transition	0.331 pN
θ_{36}	Load distribution between states 3 and 6	0.9999
ω_{41}^{0}	Forward transition from state 4 to state 1*	3.53 s^{-1}
ω_{14}^{0}	Backward transition from state 1 to state 4	4.00 s^{-1}
F_{41}	Force dependence on the 4–1 transition	1.12 pN
θ_{41}	Load distribution between states 4 and 1	0.0001
ω_{88}^{0}	The transitions from state 8 to state 8	4.00 s^{-1}
F_{88}	Force dependence on the 8-to-8 transition	2.59 pN
θ_{88}	Load distribution on the 8-to-8 transition	0.0001
ω_{77}^{0}	The transitions from state 7 to state 7	1.00 s^{-1}
F_{77}	Force dependence on the 7-to-7 transition	2.76 pN
θ_{77}	Load distribution on the 7-to-7 transition	0.0001

[*] Determined by the detailed balance condition.

Table S3. The detachment rates in the nine-state network model where state 0 is the absorption (unbinding) state

Parameters	Meaning	Values
k_{90}^{0}	Detachment from state 9 to state 0	0.550 s^{-1}
F_{90}	Force dependence on the 9-to-0 unbinding	2.76 pN
k_{20}^{0}	Detachment from state 2 to state 0	7.00 x 10^{-2} s^{-1}
F_{20}	Force dependence on the 9-to-0 unbinding	6.90 pN
k_{50}^{0}	Detachment from state 5 to state 0	0.550 s^{-1}
F_{50}	Force dependence on the 5-to-0 unbinding	2.76 pN
k_{60}^{0}	Detachment from state 6 to state 0	9.00 x 10^{-2} s^{-1}
F_{60}	Force dependence on the 6-to-0 unbinding	2.76 pN
k_{80}^{0}	Detachment from state 8 to state 0	4.00 x 10^{-2} s^{-1}
F_{80}	Force dependence on the 8-to-0 unbinding	6.90 pN
k_{10}^{0}	Detachment from state 1 to state 0	1.00 x 10^{-2} s^{-1}
F_{10}	Force dependence on the 1-to-0 unbinding	6.90 pN
k_{70}^{0}	Detachment from state 7 to state 0	5.00 x 10^{-3} s^{-1}
F_{70}	Force dependence on the 7-to-0 unbinding	6.90 pN

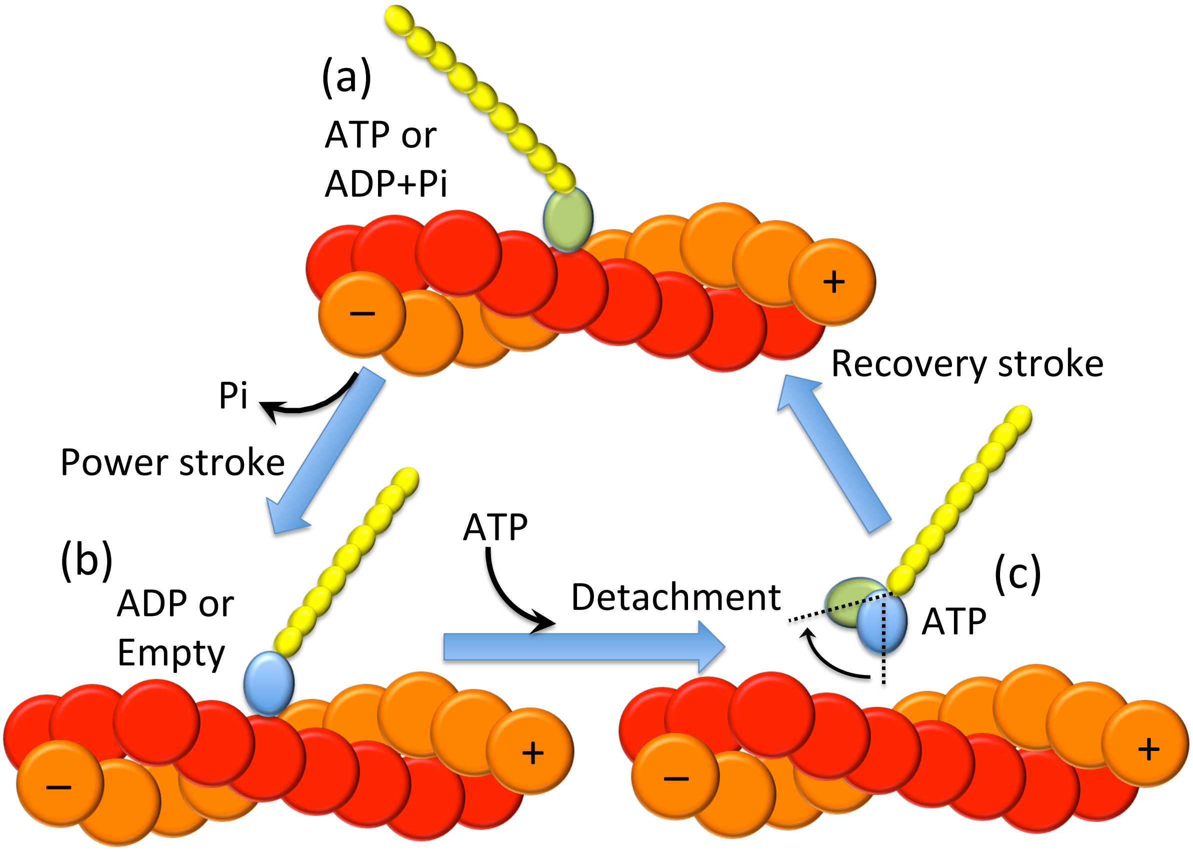

Figure S4. The head-neck angle of myosin V head[13,14]. (a) The pre-power-stroke or post-recovery-stroke conformation with ATP or ADP.Pi binding, (b) the post-power-stroke conformation with ADP binding or without nucleotide, and (c) the pre-recovery-stroke conformation with ATP binding.

References

1. Liepelt, S. & Lipowsky, R. Steady-state balance conditions for molecular motor cycles and stochastic nonequilibrium processes. *Europhys. Lett.* **77,** 50002 (2007).
2. Sumi, T. Design principles governing chemomechanical coupling of kinesin. *Sci. Rep.* **7,** 1163 (2017).
3. Seifert, U. Stochastic thermodynamics of single enzymes and molecular motors. *Eur Phys J E Soft Matter* **34,** 1–11 (2011).
4. Liepelt, S. & Lipowsky, R. Kinesin's Network of Chemomechanical Motor Cycles. *Phys. Rev. Lett.* **98,** 258102 (2007).
5. Liepelt, S. & Lipowsky, R. Impact of Slip Cycles on the Operation Modes and Efficiency of Molecular Motors. *J Stat Phys* **141,** 1–16 (2010).
6. Bierbaum, V. & Lipowsky, R. Chemomechanical Coupling and Motor Cycles of Myosin V. *Biophys J.* **100,** 1747–1755 (2010).
7. Zhang, C., Ali, M. Y., Warshaw, D. M. & Kad, N. M. A branched kinetic scheme describes the mechanochemical coupling of Myosin Va processivity in response to substrate. *Biophys. J.* **103,** 728–737 (2012).
8. Wallis, W. D. *A Beginner's Guide to Graph Theory*. (Springer Science & Business Media, 2010). doi:10.1007/978-0-8176-4580-9
9. Schief, W. R., Clark, R. H., Crevenna, A. H. & Howard, J. Inhibition of kinesin motility by ADP and phosphate supports a hand-over-hand mechanism. *Proc. Natl. Acad. Sci. U.S.A.* **101,** 1183–1188 (2004).
10. La Cruz, De, E. M., Wells, A. L., Rosenfeld, S. S., Ostap, E. M. & Sweeney, H. L. The Kinetic Mechanism of Myosin V. *Proc. Natl. Acad. Sci. U.S.A.* **96,** 13726–13731 (1999).
11. Hill, T. L. Interrelations between random walks on diagrams (graphs) with and without cycles. *Proc. Natl. Acad. Sci. U.S.A.* **85,** 2879–2883 (1988).
12. Hill, T. L. *Free energy transduction and biochemical cycle kinetics*. (Springer, 1989).
13. Sellers, J. R. & Veigel, C. Direct observation of the myosin-Va power stroke and its reversal. *Nat Struct Mol Biol* **17,** 590–595 (2010).
14. Shiroguchi, K. *et al.* Direct Observation of the Myosin Va Recovery Stroke That Contributes to Unidirectional Stepping along Actin. *PLoS Biol.* **9,** e1001031 (2011).
